# Coordinate based meta-analysis of motor functional imaging in Parkinson’s: disease-specific patterns and modulation by dopamine replacement and deep brain stimulation

**DOI:** 10.1007/s11682-019-00061-3

**Published:** 2019-02-27

**Authors:** YUE Xing, Christopher Tench, Monton Wongwandee, Stefan T. Schwarz, Nin Bajaj, Dorothee P. Auer

**Affiliations:** 1 Radiological Sciences, Division of Clinical Neuroscience, University of Nottingham, Nottingham, NG7 2UH UK; 2 Sir Peter Mansfield Imaging Centre, University of Nottingham, Nottingham, NG7 2UH UK; 3 NIHR Nottingham Biomedical Research Centre, University of Nottingham, Nottingham, NG7 2UH UK; 4 Radiological Sciences, Sir Peter Mansfield Imaging Centre, NIHR Nottingham BRC, University of Nottingham, Nottingham, NG7 2UH UK; 5 Division of Clinical Neurology, Queen’s Medical Centre, University of Nottingham, Nottingham, UK; 6 Department of Medicine, Srinakharinwirot University, Nakhon Nayok, Thailand; 7 Department of Radiology, Cardiff and Vale University Health Board, Cardiff, Wales; 8 Department of Neurology, Nottingham University Hospitals, Nottingham, NG7 2UH UK

**Keywords:** Coordinate based meta-analysis, Dunctional imaging, Parkinson’s disease, Dopamine replacement, Deep brain stimulation, Compensatary

## Abstract

**Objective:**

To investigate factors affecting the pattern of motor brain activation reported in people with Parkinson’s (PwP), aiming to differentiate disease-specific features from treatment effects.

**Methods:**

A co-ordinate-based-meta-analysis (CBMA) of functional motor neuroimaging studies involving patients with Parkinson’s (PwP), and healthy controls (HC) identified 126 suitable articles. The experiments were grouped based on subject feature, medication status (onMed/offMed), deep brain stimulation (DBS) status (DBSon/DBSoff) and type of motor initiation.

**Results:**

HC and PwP shared similar neural networks during upper extremity motor tasks but with differences of reported frequency in mainly bilateral putamen, insula and ipsilateral inferior parietal and precentral gyri. The activation height was significantly reduced in the bilateral putamen, left SMA, left subthalamus nucleus, right thalamus and right midial global pallidum in PwP_offMed_ (vs. HC), and pre-SMA hypoactivation correlated with disease severity. These changes were not found in patients on dopamine replacement therapy (PwP_onMed_ vs. HC) in line with a restorative function. By contrast, left SMA and primary motor cortex showed hyperactivation in the medicated state (vs. HC) suggesting dopaminergic overcompensation. Deep-brain stimulation (PwP during the high frequency subthalamus nucleus (STN) DBS vs. no stimulation) induced a decrease in left SMA activity and the expected increase in the left subthalamic/thalamic region regardless of hand movement. We further demonstrated a disease related effect of motor intention with only PwP_offMed_ showing increased activation in the medial frontal lobe in self-initiated studies.

**Conclusion:**

We describe a consistent disease-specific pattern of putaminal hypoactivation during motor tasks that appears reversed by dopamine replacement. Inconsistent reports of altered SMA/pre-SMA activation can be explained by task- and medication-specific variation in intention. Moreover, SMA activity was reduced during STN-DBS, while dopamine-induced hyperactivation of SMA which might underpin hyperdynamic L-dopa related overcompensation.

**Electronic supplementary material:**

The online version of this article (10.1007/s11682-019-00061-3) contains supplementary material, which is available to authorized users.

## Introduction

Parkinson’s disease (PD) is the second most common neurodegenerative disorder (de Lau and Breteler 2006). Dopaminergic deficiency, established as the major driver of motor dysfunction in People with Parkinson’s (PwP), primarily affects the nigrostriatal pathway and the striatal-cortico-thalamic circuit (Houk and Wise 1995; Rivlin-Etzion et al. 2006; van Eimeren and Siebner 2006). The symptoms of PD are caused by a dysfunction of these large-scale brain networks. A canonical pathophysiological model proposed that PD symptoms reflect hypoactivation of the direct and hyperactivation of the indirect striato-cortical pathways (Alexander et al. 1986). In PD animal models activation of the direct pathway has been demonstrated to reduce freezing, increase locomotion and prevent bradykinesia (Kravitz et al. 2010). By contrast, excitation of the indirect pathway elicits a parkinsonian-like state via relay regions including the external parts of globus pallidus and subthalamic nucleus (Purves 2012).

Over past decades, a growing number of motor-tasked neuroimaging studies have identified differences in the functional covariance pattern of regional brain activity and motor task execution in PwP from that in age-matched healthy controls. This functional reorganization of brain networks in PD has been suggested to be a combined outcome of the disease effect and the subsequent compensatory mechanisms. To compensate for failure of the normal pathways (Kalmar et al. 2011; Samuel et al. 1997; H. Yu et al. 2007), alternative motor loops are thought to be recruited in order to maintain the performance of complex movements (Mallol et al. 2007).

Although several motor functional MRI (fMRI) studies suggested that the striatum and associative motor cortex are generally involved in the functional abnormality and may participate in compensatory functional reorganization in PD, there is a lack of consistency in the activation changes described in these regions. These inconsistent results might be associated with study-wise differences in features including task paradigms, imaging acquisition and/or analysis methodologies and patient characteristics (e.g. “on” or “off” status and Parkinsonian phenotype) or variation in power. PD is characterised by a dopaminergic deficit and there is strong clinical evidence for the efficacy of dopamine replacement therapies using the amino acid L-3, 4-dihydroxyphenylalanine (L-DOPA) and dopamine agonists (Buhmann et al. 2003; Haslinger et al. 2001; Jenkins et al. 1992; Rascol et al. 1994). In addition to clinical motor improvement, the beneficial effects of dopamine replacement have been mechanistically demonstrated in a number of neuroimaging studies on movement-related brain activation (Agosta et al. 2014; Kwak et al. 2010; Nandhagopal et al. 2011; Wu et al. 2009). However, it is conceivable that not all alterations of motor brain activation observed under dopamine replacement therapy reflect the restorative effects of the treatment. The activation patterns seen may also highlight enhanced dopamine-dependent compensatory mechanisms or demonstrate the neural underpinning of adverse drug effects (Brotchie and Fitzer-Attas 2009; Fahn et al. 2004; Holloway et al. 2000; PS 2002; Zigmond et al. 1984).

A further important cause for the discrepancies of neuroimaging results could be variation in the probed motor processes, such as motor preparation and prediction for intentional actions. It is well known that PwP have difficulty with self-initiating movements, whereas external cues/marks can help improving their motor performance. In fact, studies have indicated that in externally-triggered paradigms, brain regions involved in anticipation and motor preparation were more likely to be activated due to the regularity and predictability of the incoming stimulus, whereas self-initiation or self-selected paradigms engage with a different set of brain areas that contribute to the ‘volitional action system’ (Jahanshahi et al. 1995).

Another disputed issue is the effect of alternative pathway recruitment to compensate for the depletion of striatal dopamine. More than two decades ago, a classical dopaminergic neuronal adaptive strategy was proposed, suggesting that these cells within the nigrostriatal system initiated a series of neurochemical changes to rectify the loss of dopamine-involved homeostasis caused by the disease (Bezard et al. 2003). However, several electrophysiological experiments on animals have challenged this model by showing that the motor symptoms could be restored without increasing the level of extracellular dopamine (Ponce and Lozano 2010). Therefore, it is still unclear how the motor system restores motor function after degeneration of dopaminergic neurons.

Coordinate based meta-analysis of functional neuroimaging studies allows to synthesize the imaging literature on brain disorders, to investigate common patterns and moderator factors addressing the typically low power of original studies. Problematically, early implementations of coordinate meta-analysis tools were themselves prone to false positive results. A previous meta-analysis of motor activation studies in Parkinson’s used this method and found that abnormal activation in the putamen seems to be the source of motor impairment in PD, which can be improved by dopaminergic medication. However, their meta-analysis does not provide the evidence to account for the heterogeneity in existing reported activation differences between patients and healthy controls and they concede that their proposed mode of movement selection (motor timing and selection) failed to explain this condition. In addition, the approach of their correlation analysis (activation deficiency and motor impairment) without correcting for multiple voxel-wise correlations needs to be reconsidered when interpreting the results (Herz et al. 2014a; Tench et al. 2013). In this study, we aimed to investigate the possible reasons for reported controversies of abnormal motor activation in PD. We provide a robust synthesis of evidence of PD-related alterations of motor activation that confirms the deficiency of motor activation in the left posterior putamen in PD. We also present new convergent brain imaging evidence that informs on the neural basis of motor impairment in Parkinson’s disease, involving motor intention, disease severity, and drug effects. As one of the most common deep brain stimulation approaches, the efficacy of STN DBS therapy on the clinical outcomes of motor function has been demonstrated by meta-analyses (Kleiner-Fisman et al. 2006; Perestelo-Perez et al. 2014). However, it still remains under debate of how the DBS STN exerts the improving effect on PD patients. The present meta-analysis provides additional evidence about the underlying mechanism by taking account of the medication status, stimulation side and the experimental condition (i.e. during motor task or resting). Additionally, we compared the DBS effect with the dopaminergic medication effect to investigate the commonality and difference between the two interventions.

To improve the quality of the meta-analysis we aimed for adequate homogeneity in functional results by only including upper extremity motor task studies, also assessed the role of experimental factors and used quality control tools from an improved novel meta-analysis technique in order to detect coordinate reporting errors (Tench et al. 2013). We also mirrored the coordinates from left to right based on the lateralization of the motor activity in normal HC to study the disease laterality in PD. In this study, we did not include other surgical interventions such as pallidotomy, and thalamotomy because few neuroimaging studies reported activation coordinates, preventing spatial synthesis and meta-analysis. In addition, unlike DBS which has strong and consistent clinical evidence of definite efficacy on motor impairment (other than gait kinematics) for PD, the non-invasive brain stimulations, including transcranial magnetic stimulation (TMS) and transcranial direct current stimulation (tDCS) still need to be validated in clinical settings, and thus studies applying those methods were excluded (Benninger and Hallett 2015; Shirota et al. 2016; Strafella et al. 2003a, 2006).

## Materials & methods

### Literature search and selection

We undertook a literature search of Medline, ScienceDirect and Neurosynth databases for functional Magnetic Resonance Imaging(fMRI) and/or Emission Tomography (SPECT or PET) studies involving people with idiopathic Parkinson’s disease and healthy volunteers published in English using the following keywords: “Parkinson’s disease” [Medical subject heading(MeSH)], “functional magnetic resonance imaging” Or “functional MRI” Or “fMRI”, “Positron-Emission Tomography” [MeSH], “Tomography, Emission-Computed, Single-Photon” [MeSH], Motor task or Movement task, “Deep brain stimulation” Or “DBS”. This search conducted by three independent authors resulted in 2496 studies with searching time as “All Year” till 18th January 2017.

Following pre-specified inclusion and exclusion criteria, we grouped all the experiments from healthy controls (HC), people with Parkinson’s (PwP) studied on or off medication (PwP _onMed_ or PwP _offMed_) into seven main categories (Table 1).

**Table 1:** Information of the fMRI and PET studies for different meta-analyses

Groups/contrasts	Contrasts, group details	Papers	Experiments (No.)	Extracted Coordinates (No.)	Subjects (No.)
For coordinate based aggregation meta-analysis (CBMA) and respective contrasts between groups (CMA)*					
1	Activation, PwP_offMed_	15	23	203	149
2	Activation, PwP_onMed_	5	6	37	49
3	Activation, PwP	20	29	240	198
4	Activation, HC	61	110	1406	698
For meta-analysis of contrast (MAC) ** reported between PwP vs. HC					
I vs II	Activation PwP_onMed_ vs. PwP_offMed_	7	9	30	67 PwP
II vs III	Activation, PwP_offMed_ vs. HC	26	34	383	331 PwP
I vs III	Activation, PwP_onMed_ vs. HC	4	5	44	45 PwP

### Inclusion and exclusion criteria

#### Inclusion criteria

i. subjects were either people with idiopathic Parkinson’s disease or healthy controls
ii. functional neuroimaging methods included functional MRI, PET or SPECT
iii. studies using motor task involving the upper extremities
iv. activation clusters of task versus rest (baseline) contrast were reported for single group studies (HC, PwP) or activation clusters of group differences were reported.
v. the coordinates of the activated foci were reported in either Talairach or Montreal neurological institute (MNI) standard space.
vi. studies in which deep subthalamus brain stimulation (DBS) were conducted.

#### Exclusion criteria

i. article type was not original full research article of functional imaging during motor task
ii. studies investigated motor tasks involving cognitive task during motion experiments (i.e. motor learning), visuo-motor coordination, task involving body parts outside upper extremities, gesture task specific to object, motor imagery task, and studies in which the healthy volunteers performed tasks that did not match with PD within the same articles.
iii. studies only applied and reported the results of brain functional connectivity, multivariate analyses or covariance analyses
iv. studies reporting only ROI-based analysis
v. studies in which the coordinates were not reported
vi. studies where scanning coverage does not include top of the brain and most part of the cerebrum
vii. studies which applied non-invasive brain stimulation i.e. TMS and tDCS or other surgical intervention, such as pallidotomy, thalamotomy and GPi DBS.
viii. Those with less than 5 participants were included.

### Data extraction and quality checks

Coordinates of the peak focal activations, Z(t) scores, population numbers, modality approach, and contrast (group) condition were extracted for each study (see Table S1 in Supplementary Information [SI] for more details) and entered into the database by two investigators independently and were checked by the other individual for quality control purposes. There were no discrepancies between investigators. In addition, we merged articles and removed duplicate reports that used the same subjects to prevent bias of results towards any particular population. This was carried out following a check of duplicated reported coordinates as implemented in (Tench et al. 2013) http://www.nottingham.ac.uk/research/groups/clinicalneurology/neuroi.aspx [30] .

### Coordinate based meta-analysis (CBMA)

The extracted coordinates were fed into a series of CBMA after a systematic data quality check as previously described (Tench et al. 2013). More detailed description of the method for generating activation likelihood estimates can be found elsewhere (Tench et al. 2013). To briefly summarize, each reported coordinate of significant activation as well as deactivation foci from every included publication was modelled as a spatial 3D truncated Gaussian distribution. Importantly the full width half max was automatically modified to account for the number of studies included in the analysis; this helps to prevent false positive results for large numbers of studies, and false negatives for small numbers of studies (Tench et al. 2014). As the frequency of a given focus being reported increases, the likelihood of activation enhances. We then derived all the significantly activated regions by thresholding the likelihood of activation at a value obtained from a nonparametric permutation test, corrected for multiple comparisons (Tench et al. 2013, 2014). The results of all the analyses are presented in Talairach space.

### Contrast meta-analysis (CMA) and omnibus test

To analyse spatial differences in activation pattern between groups, a contrast meta-analysis (CMA) was performed whereby coordinates from any two groups are compared using a permutation test. CMA is prone to type II error due to very stringent *p*-values after controlling for many statistical tests. Hence, we complemented CMA by an omnibus test that is more sensitive for detecting subtle differences that spread across regions of the activation pattern (Tench et al. 2014), which may be unrevealed by CMA. False positive results are controlled, using the false cluster discovery rate, at a level of 0.1 to further mitigate against the low power of CMA (Tanasescu et al. 2016).

### Meta-analysis of contrast (MAC)

Studies directly reporting contrasts between HC and patient groups lend themselves to aggregate these contrast maps (meta-analysis of contrasts [MAC]), thereby allowing to analyse both spatial differences in activation and in differences in local activation height. False positive results are controlled, using the false cluster discovery rate at a level of 0.05.

### Design of group contrasts and correlation analysis

#### Demographic and experimental factors

Prior to conducting all the meta-analyses, in an attempt to maximise power but reduce unwanted heterogeneity between study designs, we first examined potential nuisance factors from laterality (contralateral vs. ipsilateral to right-hand movement), age (≤30 years vs. ≥60 years), sex (male vs. female), and potential influence of intention-related process on motor task, including the self-initiated (SI) and externally-triggered (ET) movement. Lateralization was examined in HC and PwP groups after mirroring the coordinates obtained from motor experiments using left upper limb to the opposite side, given the majority used right upper limb, to minimize laterality effects.

#### The effect of Parkinson’s disease

One of the main aims was to identify an experimentally invariant disease specific pattern and to investigate its interrelation with disease severity. We undertook the following group and subgroup comparisons: *CMA and omnibus test were performed contrasting PwP* vs. *HC, PwP*_*offMed*_ vs. *HC, and PwP*_*onMed*_ vs. *HC*. *Meta-analysis of contrast* (*MAC) was conducted based on PwP*_*offMed*_ *> HC and PwP*_*offMed*_ *< HC*. In addition, we correlated the off-med UPDRS score as marker of the motor impairment with the standardized activation of all the significant areas. To normalize the difference of the cohort size, which has not been taken into account in the previous analysis (Herz et al. 2014a), we divided the reported Z scores of each coordinate by the square root of the subject number. Z values of studies that did not contribute to the respective regions were removed. Then we applied a weighted linear regression analysis and the outcomes of the correlation between the Z scores and UPDRS-III scores were thresholded at *P* < 0.05.

#### The effect of dopamine replacement therapy

For assessing the dopaminergic medication effect, we investigated PwP_onMed_ vs. PwP_offMed_, PwP_offMed_ vs. HC, and PwP_onMed_ vs. HC using CMA. We also performed MAC analysis on the subgroup comparison PwP_onMed_ > PwP_offMed_, PwP_onMed_ < PwP_offMed_, PwP_onMed_ > HC, and PwP_onMed_ < HC.

#### The effect of deep brain stimulation

To better understand the therapeutic mechanism underlying deep brain stimulation in PD, we synthetized the neuroimaging evidence on deep brain stimulation. Due to the limited number of reported articles with spatial coordinates in the effect of globus pallidus DBS (GPi DBS) on motor function in PD, we only included studies about STN DBS. We compared DBSoff vs. DBSon during hand movements, DBS effect during two experimental conditions (during motor task vs. resting) on account of medication status (off medication), and stimulation side.

#### The effect of deep brain stimulation versus dopamine replacement therapy

To investigate the difference between the effects of two treatments, we compared PwPonMed vs PwPoffMed and DBSon vs DBSoff using CMA analysis and omnibus test.

## Results

**Fig. 1:**
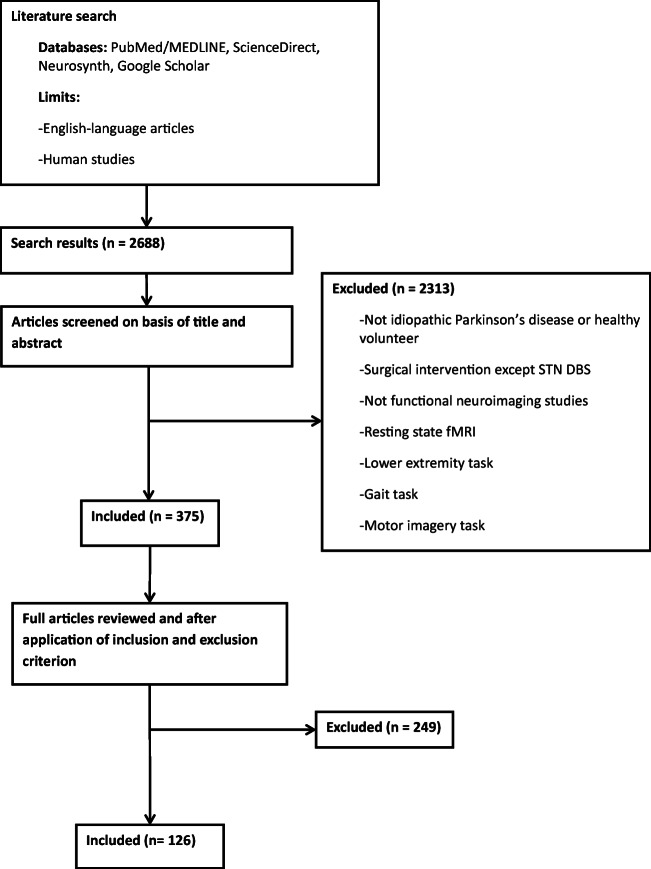
Flowchart of functional MRI and PET studies recruited in the analysis

After primary screening, our first search identified 375 articles, of which 126 studies were included, involving 1024 HC, and 657 PwP (222 motor task experiments) (Fig. 1, Tables 1 and 2). PwP had an average reported UPDRS motor score of 24.7 (±9.1 standard deviation), reported Hoehn and Yahr scale median: 1.8(interquartile range, 1) and disease duration ranging from 1.8 to 12.9 (mean age ± standard deviation, 6.6 ± 3.6) years. The mean age of the HC included in this study was 35.5 years (±16.0, standard deviation) with a subgroup of elder HC (mean age, 64.0 ± 2.6 years).

**Table 2:** Meta-analysis of group contrasts (MAC) using activation likelihood estimates

Hemisphere	Brain region	Brodmann Area	Coordinates (x y z)	*P* value
		*PwPoffMed < HC*		
Right	Lentiform Nucleus	Putamen	21.2 5.6 5.2	0.000195
Left	Medial Frontal Gyrus	BA 6	−6.0 -18.0 51.0	0.001314
Left Brainstem	*	Subthalamic Nucleus	−10.7 -13.7 -3.4	0.000464
Left	Lentiform Nucleus	Putamen	−23.3 -2.4 11.8	0.000645
Right	Thalamus	*	13.8–5.6 4.0	0.000607
Right	Lentiform Nucleus	Medial Globus Pallidus	16.0–4.4 1.6	0.00156
		*PwPonMed>* *PwPoffMed*		
Left	Superior Frontal Gyrus	BA 6	−16.0 16.0 58.0	0.004158
Right	Middle Frontal Gyrus	BA 6	32.0 0.0 50.0	0.005447
Right	Lentiform Nucleus	Putamen	24.9–3.8 4.3	0.005395
Left	Lentiform Nucleus	Putamen	−25.1 -3.5 3.5	0.002026
Left	Claustrum	*	−32.5 -10.9 2.7	0.008842
		*PwPonMed > HC*		
Left	Medial Frontal Gyrus	BA 6	−3.6 -6.8 55.8	0.006447
Left	Precentral Gyrus	BA 4	−31.4 -22.1 59.3	0.003342
Left	Precentral Gyrus	BA 6	−35.1 -12.6 58.3	0.008474
		*PwPonMed < HC*		
Right	Postcentral Gyrus	BA 2	27.7–33.8 61.0	0.0017
Right	Cerebellum	*	21.4–47.9 -25.1	0.00632

### Potential demographic or experimental factors affecting the motor activation pattern

No significant age difference was found using the unpaired t-test, but there was a marked difference between the HC in PD-related studies and those from articles that simply investigated the brain activation pattern in HC during motor tasks (*p* < 0.05 based on omnibus test). This allowed us to examine the normal aging effect on motor task for healthy volunteers. However, we failed to observe regions of significant differential activation between young HC and elderly HC during the motor performance with our CMA analysis when we selected studies with population of age above 60 years (elderly) versus those under 30 years (young) as the criteria. Nevertheless, we were still able to illustrate the frequency of the regions, such as bilateral SMA, right premotor cortex, left insula and right cerebellum that showed significant contribution (*p* < 0.05) to the between-group difference (see Fig. S4 in supplementary material). Furthermore, we used MAC on three articles that focused on the age effect on brain activation during motor execution, but none of the reported age-dependent clusters were found to be consistent across the three studies.

Neither did we find significant differential patterns with regard to sex or the “what” component of intentional action (i.e. what movement or sequence did the participants choose). However, a few regions were labelled to be highly involved in “when”- self-selecting the moment of execution or not using MAC (Fig. S1). We hence pooled all data, but examined the effect of movement intention by group separately.

### Spatial pattern of motor activation in PwP and healthy controls

**Fig. 2:**
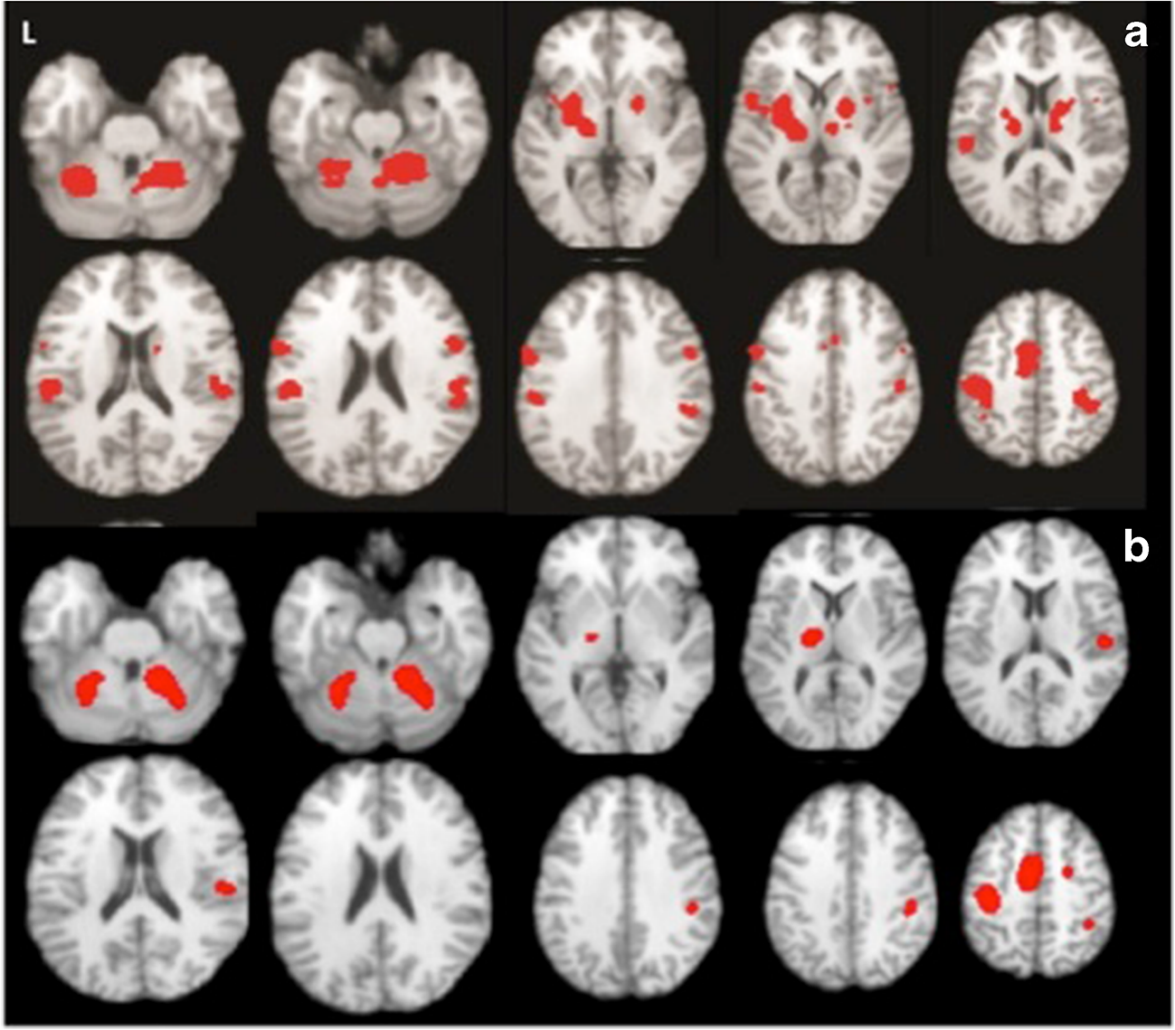
**a** Aggregated upper extremity motor-related activation pattern in healthy controls (n=686 subjects, n=109 experiments, FCDR corrected p<0.05) and **b** in people with Parkinson’s using CBMA color-coded according to medication state (red: on medication, n=49 subjects, n=6 experiments, FCDR corrected p<0.05, green: off medication, n=138 subjects, n=22 experiments)

CBMA demonstrated a consistent activation pattern in healthy controls performing motor tasks of the right upper extremity or looking at flipped results in those few studies deploying the left upper extremity (Fig. 2a). The pattern is largely bilateral involving the extended motor network with some contralateral lateralization. A similar albeit more reduced pattern was observed in PwP (Fig. 2b). Visually apparent differences in pattern expression can also be seen in the frequency maps (Supplementary material, Fig. S2), and using the omnibus test, we found that the global activation pattern did exhibit a significant between group difference (*p* = 0.0043). For instance, the HC showed more frequently reported activation in the putamen, insula, thalamus, medial frontal gyrus and inferior parietal lobule in both hemispheres, superior parietal gyrus in the left hemisphere (Fig. S2), but a lower frequency of reported activation in the right middle frontal gyrus. Contrast of meta-analysis (CMA) did not however show consistent areas of differential activation likelihood between PwP and HC.

### Disease and medication status specific brain activation contrast

**Fig. 3:**
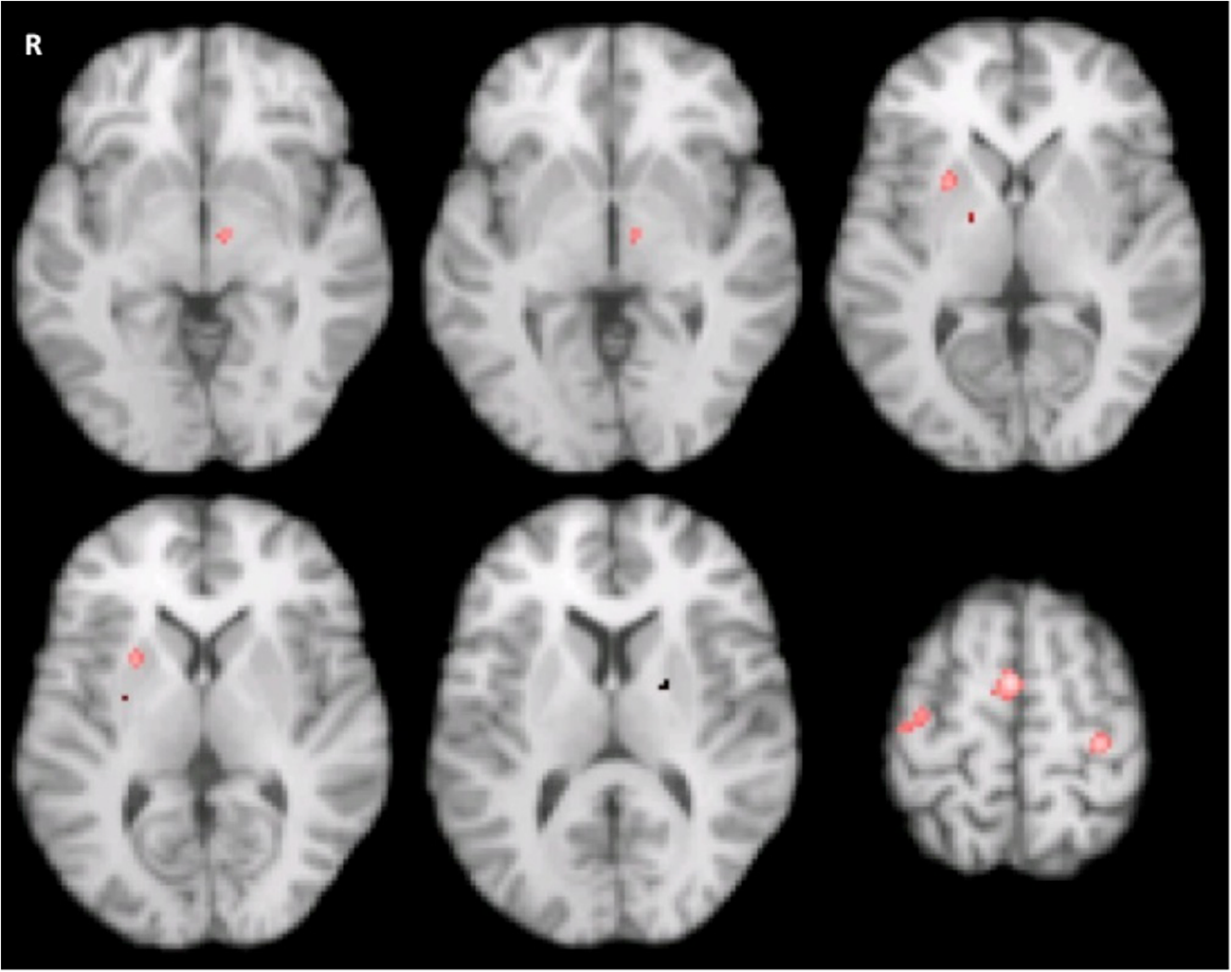
Reduced motor activation in PwP off medication vs. healthy controls (Meta-analysis of contrasts, n=34 experiments, n=331 patients vs. 327 controls, FCDR corrected p<0.05)

The inability to observe specific differences in activation likelihood between patients and controls may have resulted from heterogeneity caused by medication status. Prior to performing all the analyses, an omnibus test was used to check whether the brain activation patterns are different when left vs. right hand was used in the motor tasks. The omnibus test showed no difference between these two conditions. We then merged all the studies to increase the sensitivity of detection. To formally probe any difference caused by the disease when taking the medication status into account, we also performed a CMA HC vs. PwP_offMed_. Though no difference of activation likelihood was revealed, the omnibus test showed significant differences of global activation pattern (*P* = 0.03). Moreover, MAC found that PwP off medication displayed consistently reduced activation strength compared to HC in the bilateral putamen, left subthalamic, left medial frontal gyrus and right thalamus (*p* < 0.002) (Fig. 3 and Table 2) when undergoing a motor task.

### Correlation between brain activation and motor impairment in PwP_offMed_

**Fig. 4:**
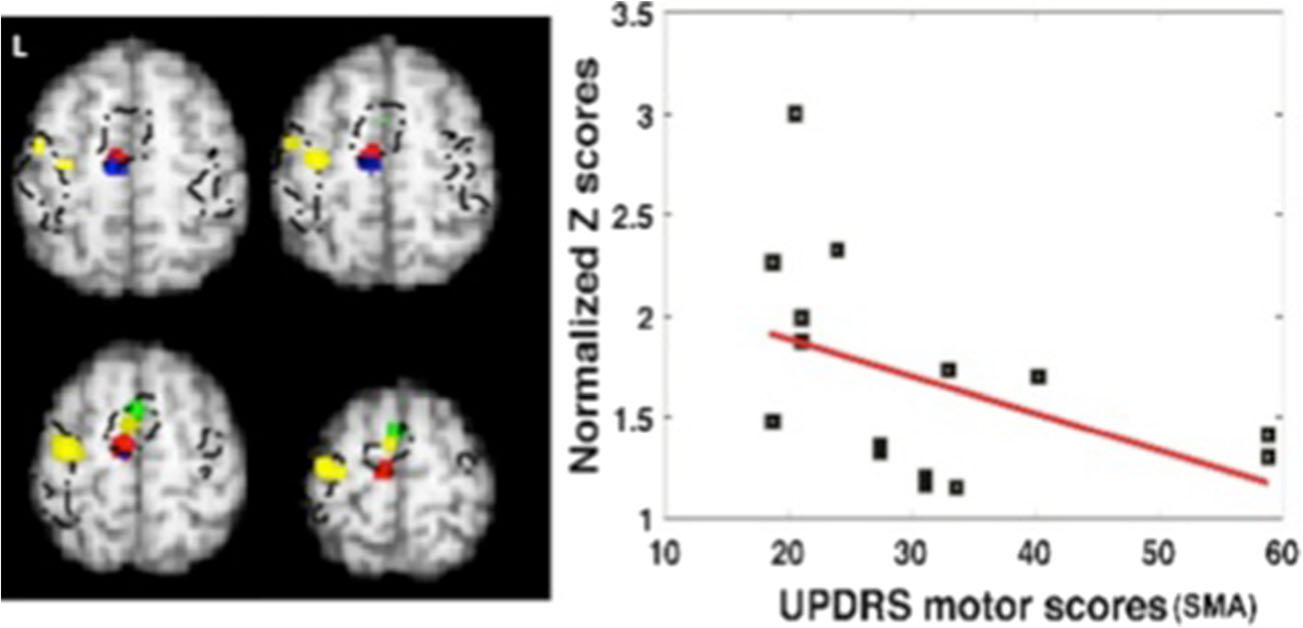
Left, The co-localization of effects from modifying factors of SMA/medial frontal gyrus motor activation in PD. Clusters were thresholded for the sake of illustration. Blue: hypoactivation due to disease effect (PwPoffMed < HC); Red: hypoactivation in DBS on phase (DBSon < DBSoff); Green: hyperactivation in self-initiated movement in PD (SI>ET); and Yellow: hyperactivation due to dopaminergic medication (PwPonMed>HC) superimposed with black dotted outline of the medial frontal activation obtained from motor activation in controls. Right, Decreased left medial frontal motor activation with increasing motor severity (Weighted meta-regression of UPDRS-III score and standardized Z scores of motor activation on PwPoffMed, p<0.05)

We investigated the relationship between the estimated local activity and severity of motor impairment using weighted meta-regression based on reported mean UPDRS-III score in 22 (17 fMRI, 5 PET PwP_offMed_) studies. Analysis was limited to significant clusters for all PwP_offMed_ vs. HC contrasts listed in Table 2. The motor score was found to be negatively correlated with the likelihood of activation in the left SMA (see area outlined by black dots in Fig. 4 left) only (rho = −0.627; *p* = 0.044; Fig. 4 right), suggesting that this regional activation decreases in PwP_offMed_ as the motor impairment increases.

### Effect of dopaminergic medication on motor activation in PwP

**Fig. 5:**
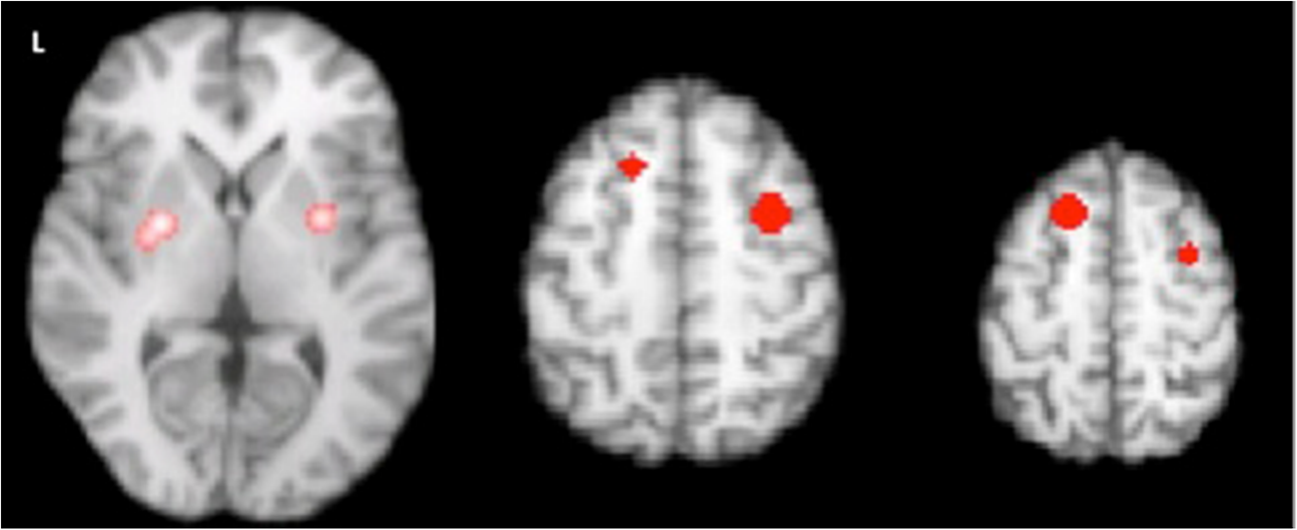
Increased contralateral and decreased ipsilateral motor activation in medicated PwP (Meta-analysis of contrasts; A: PwPonMed < HC, B: PwPonMed >HC, 5 experiments, patients n=45 vs. 44 controls, FCDR corrected, P<0.05)

To aggregate the direct within-subject effect of dopaminergic treatment, MAC comparing PwPonMed with PwPoffMed was performed. We identified several regions with motor activation consistently augmented by dopaminergic medication (PwPonMed > PwPoffMed): left superior frontal gyrus, right middle frontal gyrus, bilateral putamen and left claustrum (Fig. 5 and Table 2). There were no regions with reduced activation during the on medication state (PwPonMed <PwPoffMed).

**Fig. 6:**
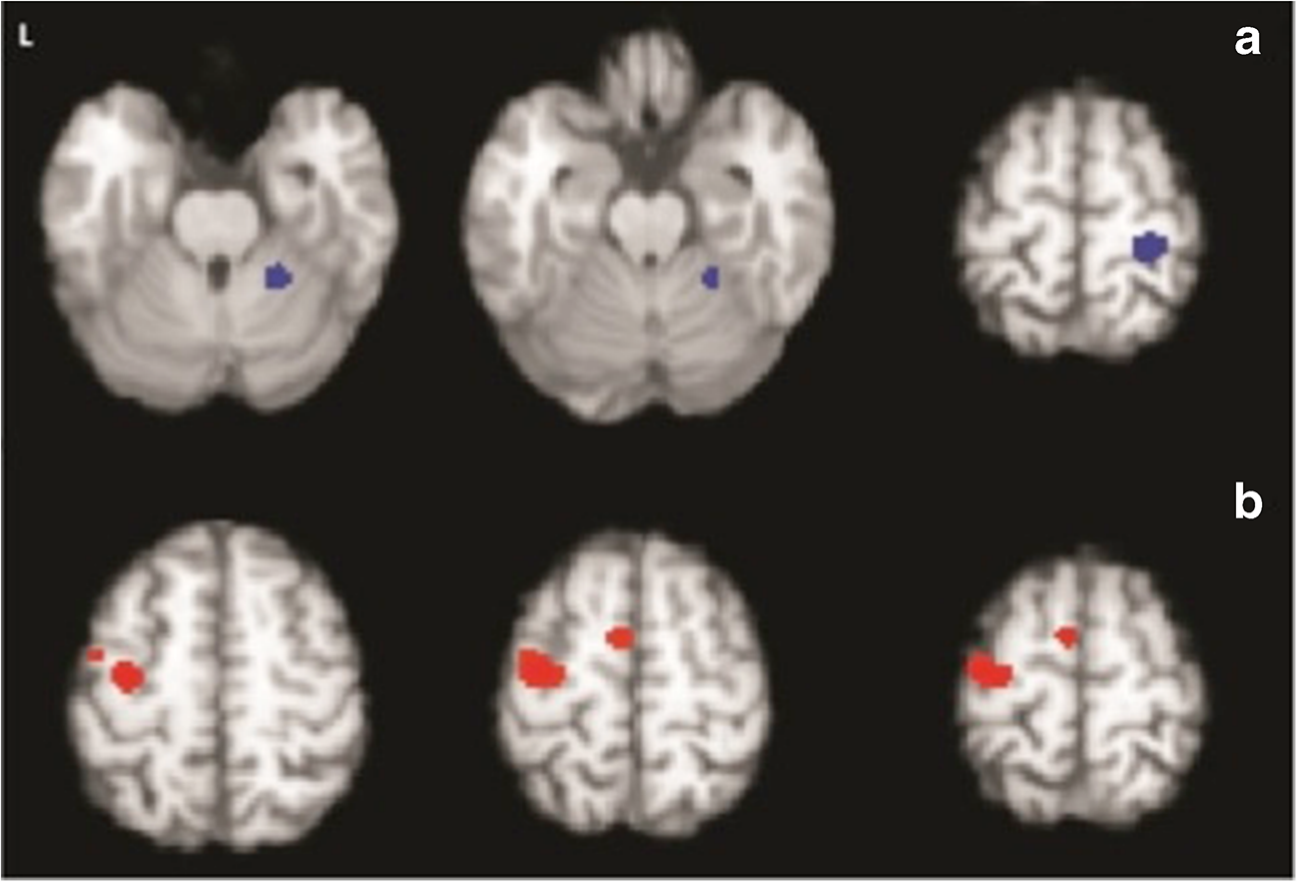
Increased motor activation due to dopamine-replacement in PwP (Meta-analysis of contrast on medication > off medication state, 9 experiments, and patients n=67, FCDR corrected, P<0.05)

To assess any possible additional effect of dopaminergic medication on brain activation, we also undertook contrasts between PwPonMed vs. HC using omnibus tests, CMA and MAC. The omnibus test supported an overall activation difference between groups (*P* = 0.04), but CMA did not reveal significant spatial differences of activation likelihood. However, MAC revealed consistent areas of both decreased and increased motor activation strength in medicated patients: PwPonMed < HC contrast showed decreased activation in the right postcentral gyrus and right anterior lobe of cerebellum (*p* < 0.05) (clusters in blue in Fig. 6 and Table 2); PwPonMed > HC contrast found increased activation in the left precentral gyrus and medial frontal gyrus (clusters in red in Fig. 6 and Table 2).

### Effect of deep brain stimulation on brain activation with off medicated PwP

**Fig. 7:**
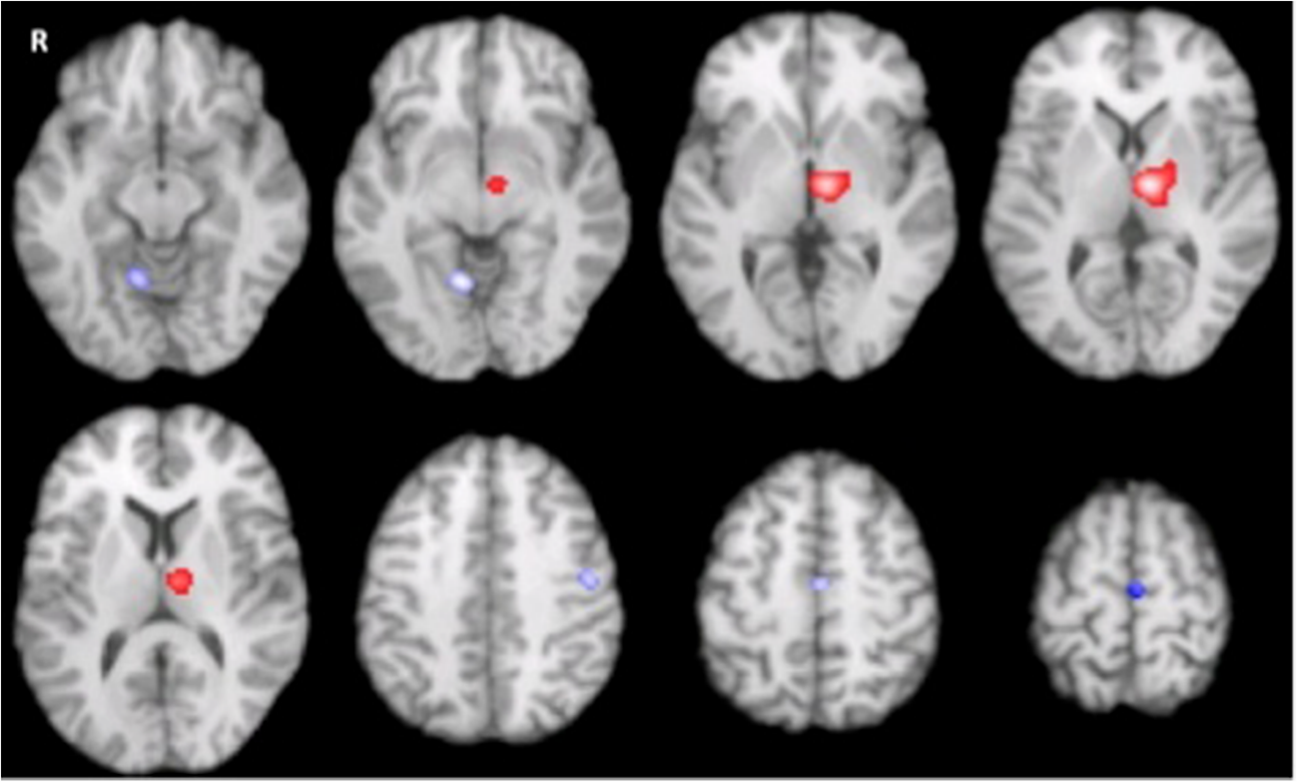
Increased left thalamus and decreased left primary motor cortex and SMA in off medicated PwP regardless of hand movement during high frequency bilateral STN DBS (Meta-analysis of contrasts; Red: DBSon > DBSoff, Blue: DBSon < DBSoff, FCDR corrected, P<0.05)

Our MAC analysis results on brain activation triggered by STN DBS was not different between hand movement and at rest. Even the most sensitive omnibus test did not show any difference due to movement status. Also, there was no effect of DBS (DBSon vs DBSoff) during hand movement. Therefore, to increase the sensitivity of detecting the potential effect of DBS, we combined all the studies regardless of the movement condition, and we revealed the main effect of the bilateral STN DBS as an increased activation of left thalamus (i.e. ventral lateral nucleus), and left globus pallidus (DBSon vs DBSoff). Compared with the DBS-off condition, activity in the left SMA, and M1, and the right culmen were reduced (Fig. 7 and Table 3).

**Table 3:** Meta-analysis of group contrasts (MAC) for STN DBS using activation likelihood estimates

Hemisphere	Brain region	Brodmann Area	Coordinates (x y z)	P value
		*DBSon > DBSoff*		
Left	Thalamus	*	−11.0 -12.0 0.0	0
Left	Thalamus	Ventral Lateral Nucleus	−10.0 -14.0 4.0	0
Left	Thalamus	Ventral Lateral Nucleus	−13.0 -15.0 6.0	0
Left	Lentiform Nucleus	Lateral Globus Pallidus	−18.0 -10.0 6.0	0.000023
		*DBSon < DBSoff*		
Left	Medial Frontal Gyrus	BA 6	−7.0 -14.0 51.0	0.003169
Left	Precentral Gyrus	BA 4	−50.0 -12.0 41.0	0.001468
Right	Culmen	*	4.0–60.0 -8.0	0.002331

### Difference between effect of deep brain stimulation and dopaminergic replacement therapy on brain activation

No consistent areas of different activation likelihood were revealed when comparing the two therapies using CMA or MAC, but there was a marked difference between the brain activation patterns in those two approaches during motor tasks (*p* < 0.05 based on omnibus test).

### Self-initiated versus externally triggered movements in Parkinson’s disease

CMA showed that external-paced motor task did not differ in the brain activation pattern from self-paced tasks in all the single groups, whereas in PwP_offMed_ > HC the supplementary motor area was consistently enhanced in the condition of self-paced task (Fig. S3).

## Discussion

Using coordinate based meta-analysis, we report consistent spatial activation patterns, but differential activation contrasts in a large group of healthy controls and people with PD and its moderation by current treatment strategies. We provide insight into the motor network alteration induced by Parkinson’s disease, its partial restoration by dopaminergic treatment, modulation by DBS and overcompensation resulting from medication.

### Neural activation pattern in simple upper extremity motor tasks in controls

Regions including primary and supplementary motor and premotor cortex, bilateral putamen, medial frontal gyrus, STN, thalamus, and medial globus pallidus (GPi) in both hemispheres as well as anterior bilateral cerebellar hemispheres were seen in our aggregated activation pattern in HC. This co-activation pattern involves the known cortical motor network and subcortical hubs from both the direct and indirect pathways, thus conforming to simultaneous deployment even during simple motor tasks, and independent from external or internal action triggering.

### Disease specific network alterations of basal ganglia system in PD

The aggregated motor co-activation pattern in PwP did not reveal consistent areas of altered activation likelihood in the whole group or in the off medication subgroup. However, the activation strength assessed by aggregation of reported contrasts was consistently reduced in the bilateral basal ganglia (i.e. putamen), left medial prefrontal cortex (SMA), left subthalamic nucleus, right thalamus and right globus pallidus. This is only partially in line with the previous meta-analysis, which demonstrated increased activation in left superior parietal lobule and decreased in right putamen. Our results imply that the motor activation response of the left SMA and putamen of both hemispheres is reduced in the hypodopaminergic state. We did not find evidence for compensatory hyperactivity of motor circuitry to mitigate the loss of motor activation. This hypothesis may be further evidenced by the lack of correlation between putaminal activation and disease severity, which is again different from the previous meta-analysis (Herz et al. 2014a). This, however, is well in line with evidence from previous clinical and dopaminergic imaging findings, showing that dopamine in the putamen must be degraded to approximately 50–60% of the normal level before patients are diagnosed, and that loss of dopaminergic neurons might be complete during early disease stages resulting in a ceiling effect during later disease stages (Asanuma et al. 2006; Bruck et al. 2006; Nurmi et al. 2001; Politis 2014). Consequently, this may make the task-related activity of the whole putamen a less sensitive measure to reflect the motor impairment, whereas other non-dopaminergic structures, such as SMA correlated more strongly with clinical motor scores (see more explanation in the section associated with the role of SMA).

When interpreting the observed impaired neurovascular activation against predictions of the classical model of hyperactive indirect and hypoactive direct motor networks, a number of explanatory and moderating regional and global factors have to be considered. Disease-related regional changes in brain activity at rest are known to affect task-induced BOLD activity and high baseline activity may result in reduced motor-induced activation. Additionally, without access to concurrent motor task performance data, we cannot assess to which degree the changes of motor activation reflect differences in task performance. Previous studies showed enhanced ROI-to-ROI functional connectivity between SMA and putamen based on a resting-state fMRI in off-medication patients (Kwak et al. 2010; R. Yu et al. 2013). Hypoactivity of the putamen and motor-related cortex (i.e. SMA) may provide additional evidence by showing that their increased connectivity may be attributed to the decreased activation of both regions. Our results may well reflect hypodopaminergic changes of the motor circuit, which involves corticostriate projections from the premotor cortex to the putamen.

Our results also showed significantly reduced activation in the basal ganglia pathways, such as the globus pallidus, subthalamic nucleus and thalamus, suggesting that these regions are involved in the pathological process. Despite the fact that M1 did not show significantly decreased activation, it showed nominally higher reported frequencies than for the rest of the brain (also see Fig. S3). This may imply an alternative and more parsimonious explanation: the observed unidirectional alterations in PD can be seen to add to the critical evaluation of the classical model of basal ganglia circuits, that led to a more advanced notion that motor-related behavior is a result of a complex interaction between these two pathways, suggesting that network integration of all basal ganglia circuits subserve co-ordinated motor activity (Calabresi et al. 2014; Jahanshahi et al. 2015). Recent studies have demonstrated a hyperdirect cortico-subthalamo-pallidal pathway, which has been viewed as the quickest approach for accessing motion output. It has been shown to be operating via a complicated combination of suppression, excitation and disinhibition (Jahanshahi et al. 2015; Nambu et al. 2002). Therefore, along with the mounting pathophysiological evidence (Bergman et al. 1994; Goldberg et al. 2002; Hammond et al. 2007; Wichmann et al. 2011), the presented imaging evidence suggests that the motor deficits in PD may be better explained by the inability to activate the striato-cortical network than by simple hyperactivation of the indirect pathway.

### The dopaminergic restorative pathway in Parkinson’s disease

Dopaminergic drugs are known to play a critical role in the functional remapping of the brain during motor execution. Compared to the number of studies on PwP off-medication, fewer studies reported the on-medication condition to avoid the confounding effect of drugs (see Table S3 in suppl material for the types of DRT). However, these studies offered us the possibility to assess the differential brain activation effect of dopaminergic medication. Our meta-analysis (PwP_onMed_ > PwP_offMed_) revealed that dopaminergic medication significantly increased motor activation in the superior and middle frontal gyri. We also found increased activation in the bilateral putamen-related activation, demonstrating that dopamine induces frontal hyperactivation and largely restores the deficient motor activation of the putamen in reported cohorts of PD. This effect partially contradicts a previous meta-analysis reporting unilateral putaminal activation but no cortical hyperactivation due to dopamine replacement (Herz et al. 2014a). The lack of previous observation of this effect can be explained by a larger number of included studies and an improved meta-analytic methodology in our study. Hyperactivation of the middle frontal gyrus has been proposed to be related with the attention networks, by switching attention between the endogenous controlling processes and external stimulus (Corbetta et al. 2008; Japee et al. 2015). The amplified activation in this region indicates the drug effect may restore and strengthen the attentional process to facilitate the role of superior frontal gyrus in coordination with the execution of sensory system.

### The functional role of basal ganglia subregions in PD and region-specific effect of dopamine replacement

**Fig. 8:**
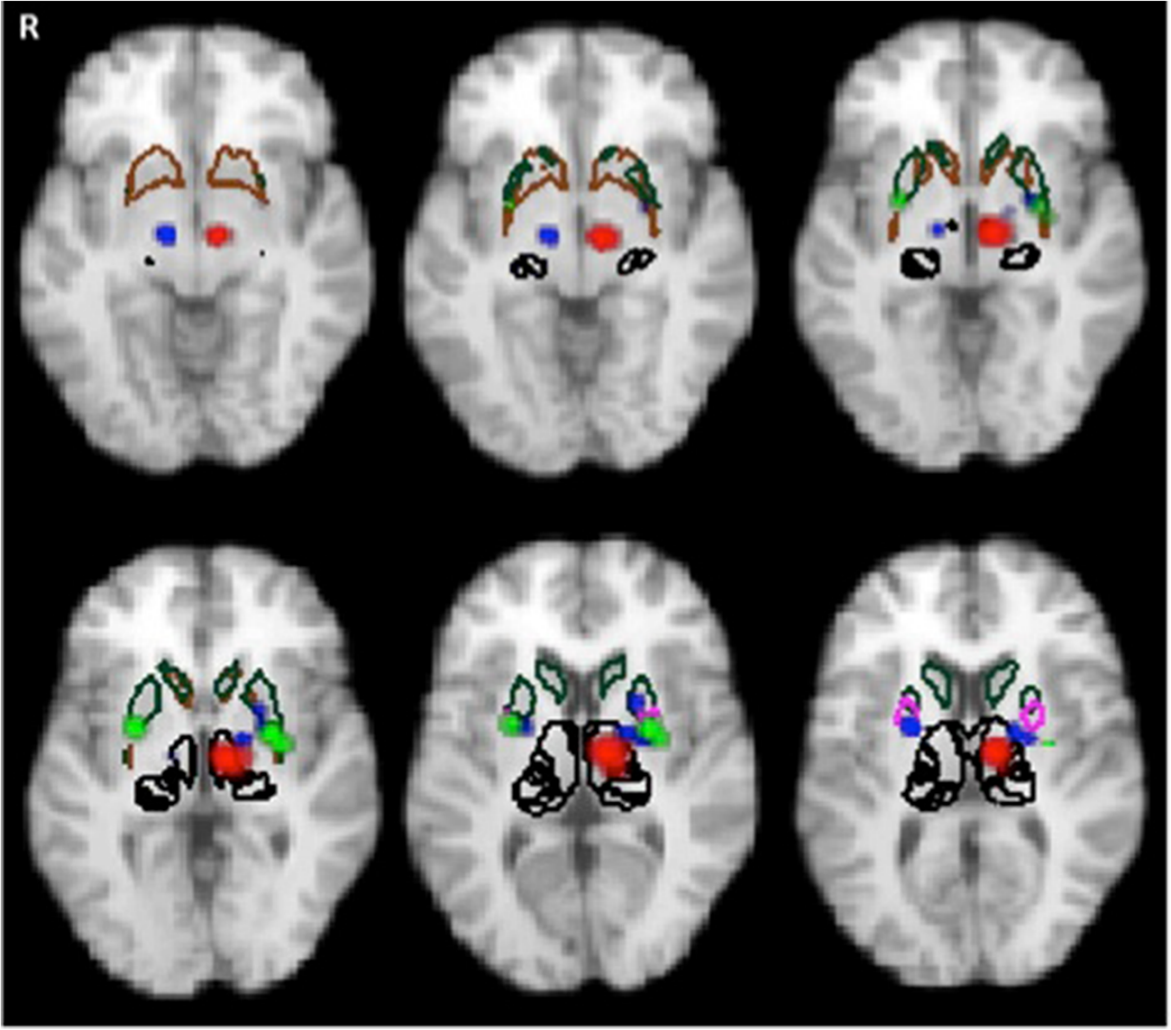
Co-localization of altered subcortical motor activation due to disease (blue: hypoactivation PwP_offMed_<HC) and STN DBS effects (green: hyperactivation DBS off < DBS on) overlain on structural connectivity atlas. The boundaries of executive (green), limbic (brown) and sensorimotor (pink) are delineated. Thalamic nuclei are outlined in black. Noticeably, no activation difference was shown in the striatal regions posterior to the dopaminergic treatment (PwP_onMed_ > PwP_offMed_)

To further discern the functional deficiency and the restorative localization in PwP, we overlaid our detected activation clusters for disease on a striatal atlas based on probabilistic diffusion tractography defining limbic, executive and sensorimotor subregions (Tziortzi et al. 2014). This illustrates that most of the deactivation areas are linked to the executive (dark green in Fig. 8) and sensorimotor cortex (pink in Fig. 8) in PD. As expected, the limbic basal ganglia subregions were least affected. It is worth noting that dopamine replacement restored the disease-related reduction of activation in the putaminal subregion that connects to the executive and motor cortex. This conforms to the clinical observation of improved motor function after dopaminergic treatment (Buhmann et al. 2003; Haslinger et al. 2001; Jenkins et al. 1992; Rascol et al. 1994). These findings are also supported by recent resting state fMRI studies showing that spontaneous activity in the posterior putamen was uniquely linked to cortical motor activity, while the anterior part appears more connected to the pre-supplementary motor area and anterior cingulate cortex (Hacker et al. 2012; Helmich et al. 2010). Moreover, previously shown diminished functional connectivity between the posterior putamen and inferior parietal cortex in PD patients is consistent with our finding of co-deficiencies in putamen and inferior parietal lobule based on frequency maps (Fig. S2). Furthermore, our result of predominant posterior putaminal restoration of dopamine replacement in PD is supported by molecular imaging such as 18F-DOPA PET that mainly link the dorsal caudal putamen with the decline of dopaminergic function (Fearnley and Lees 1991).

### The dopaminergic effect on spontaneous brain activity in PD

Unlike the dopaminergic effect on motor activation, performing a meta-analysis of the drug effect on brain spontaneous activity remains hampered by the heterogeneity of analytical procedures and methods, and the lack of coordinate’s details and decent number of articles. For instance, the evidence of the direct influence of DRT on cerebral perfusion at resting-state is still relatively scarce and inconsistent. Nevertheless, a very recent observation of cerebral blood flow (CBF) using MRI arterial spin labelling technique showed hypoperfusion in the inferior frontal gyrus in PD after dopaminergic administration, similar to the findings from a previous PET study (Berding et al. 2001; Lin et al. 2016). Other regional CBF changes have been also identified in striatal-thalamo-cortical circuits (Hershey et al. 2003; Hirano et al. 2008; Kobari et al. 1995). But no confirmed correlation between daily uptake dose of Levodopa and hypoperfusion has been provided so far (Liepelt et al. 2009). On the other hand, despite those reported focal CBF changes, global CBF at rest is not altered significantly as a consequence of medication (Hershey et al. 2003; Hirano et al. 2008; Jenkins et al. 1992; Melamed et al. 1986). However, using the more advanced analytical approaches, including seed-based, network-based, graph-based analyses, and effective connectivity with resting-state fMRI, investigators showed that DRT might modify the intrinsic functional disorganization of the brain via the sensorimotor network and cortico-striato-thalamic network [see review (Tahmasian et al. 2015)].

### The effect of deep brain stimulation (STN DBS) on motor activation

This present meta-analysis for the first time investigated the mechanistic imaging studies to explore the role of pathway changes both at rest and under manual activation. But no differential effect of DBS was unveiled comparing the brain activation patterns in those conditions. Nevertheless, by combining all the STN DBS results together regardless of hand movement, we compared the DBSon vs DBSoff, which showed hyper-activation in the left thalamus and GP, and hypo-activation in the left SMA, M1 and cerebellum in PwP_off-med_ with indications for the efficacious implementation of DBS, which is in line with the classical indirect basal ganglia pathway. This suggests a predominantly down-regulatory cortical effect of STN DBS, and this may restore cortical dysfunction via inhibiting the pathological and/or side effect of pharmacological over-activation. Surprisingly, we did not observe significant changes of brain activation in some expected regions within the basal ganglia thalamo-cortical motor circuit, such as the putamen and/or substantia nigra, which again may imply a more complicated interactive basal ganglia network. However, a simple dilution effect cannot be excluded since we merge the studies regardless of the movement status to increase the statistical power. Therefore, more evidence with advanced analysis (i.e. functional connectivity) is needed to further tackle this question.

### Commonalities and dissociations of the effects of deep brain stimulation (STN DBS) and DRT on motor activation

**Fig. 9:**
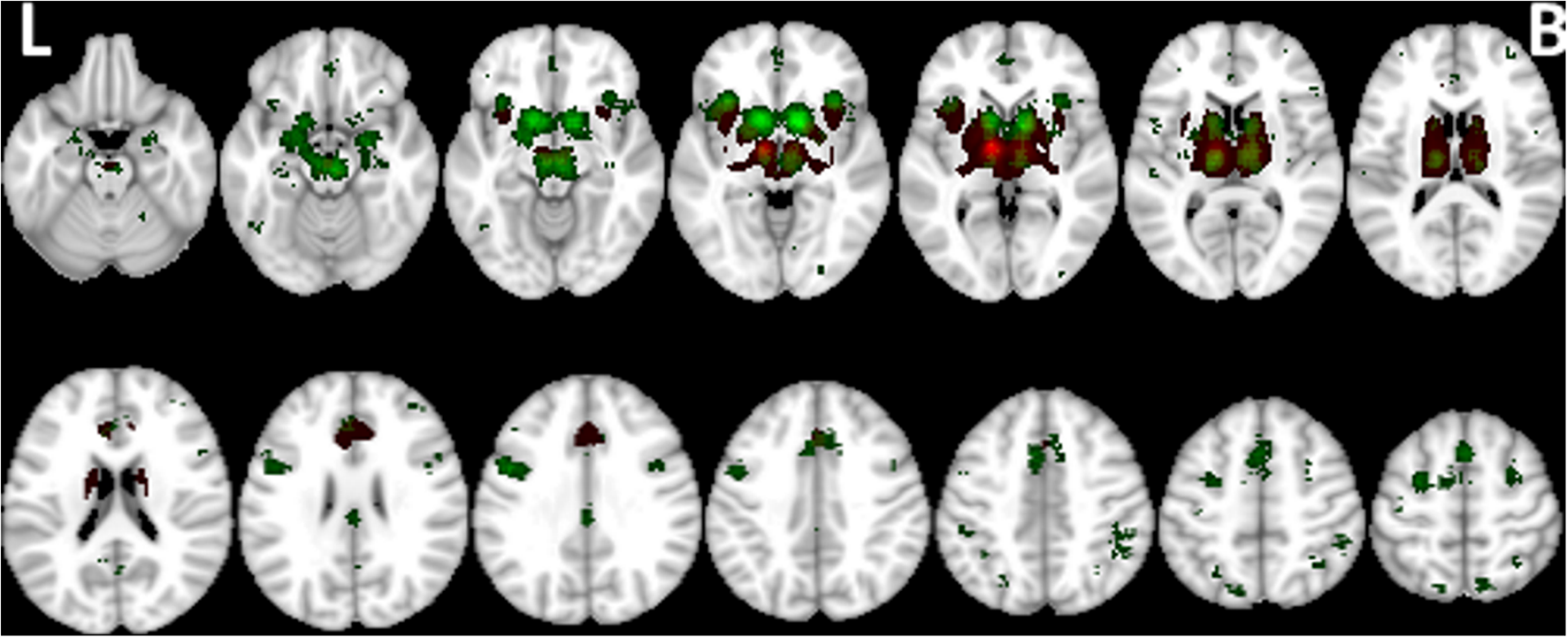
The functional connectivity from STN and dopaminergic pathway obtained from neurosynth (http://www.neurosynth.org/analyses/). Green: dopaminergic pathway; and red: undirected functional connectivity results based on the seed in subthalamus

Direct stimulation in the deep brain nuclei is an alternative to levodopa treatment for modulating the abnormal STN activity with evidence shown in improving the motor symptoms. The brain oscillation studies also showed that this improvement of motor performance might be related to the increased frequency of beta-burst induced by both levodopa and adaptive DBS (Tinkhauser et al. 2017a, b). Fig. 9 illustrates the functional connectivity from STN and dopaminergic pathway obtained from neurosynth (http://www.neurosynth.org/analyses/). Our omnibus test showed a significant difference between the activation patterns between the effects induced by the two treatments. Interestingly, our meta-analysis on STN DBS effects demonstrated increased activity in the thalamus and GP, and decreased activity in motor regions, whereas up-regulation in frontal gyri and the putamen was illustrated due to drug effect. In contrast to the direct increase of putaminal activation induced by the DRT in line with dopamine increase, the evidence for DBS highlights a restorative mechanism for the neuromodulation that targets the deficiency in the striatal-thalamic circuit in PD. This is consistent with the previous physiological finding that STN-DBS does not increase dopamine production (Benazzouz et al. 2000) although animal models demonstrated accelerated firing rate in SN (Hilker et al. 2003; Nakajima et al. 2003; Strafella et al. 2003b). Taken together, dopaminergic pharmacotherapy and STN DBS may achieve therapeutic efficacy via manipulation of different regions in similar networks (Eidelberg 2009). But this difference could also be caused by the different characteristics of patient populations, since the PwP who underwent STN DBS were more advanced with either motor fluctuation or LID, which may not be simply imposed by the loss of dopamine (Hammond et al. 2007). This abnormal SMA in the DBS-on condition, therefore, may imply an inextricably reorganized network related to both the external electrical stimulation and disease.

In addition to the dysfunction of the basal ganglia-thalamic pathway, changes in the cerebellum have been reported in PwP when DBS was on. In our meta-analysis, both HC and PD demonstrated cerebellar activation (Fig. 2a and b) and we failed to find a significant pattern difference in both cerebella in PwP_offMed_ compared to HC. Yet, when we examined its differential activation for PwP_onMed_ versus HC (PwP_onMed_ < HC), the anterior lobe of the right cerebellum showed a significant activation defect in patients after medication. Besides the variation of studies recruited for the two groups, one possible explanation for these findings might be that the cerebellum was not activated as much in the on-medication as it was in the off-medication condition in order to compensate for the drug-induced effects. This may also be supported by the suppressed activation of the culmen in PD off-med when DBS is efficacious.

### The cortical mechanism for impaired sensorimotor integration in PD

Besides the subcortical areas and cerebellum, our MAC also demonstrates activation differences in a collection of cortical regions in PD. For instance, different from the fact that no cortical region was localized in previous analysis (Herz et al. 2014a), we identified consistent left SMA hypoactivation in PwP_offMed_ and increased activation in the same region following medication (PwP_onMed_ > HC), but reduced activation when STN DBS was on. This may imply that the restoration of cortical premotor activation in PD may be a combination of the increased activation during movement and a reduced activity when less voluntary movements are involved. Discrepant from these findings, Herz et al. showed hypoactication of left M1 in PwP_onMed_ < HC, while we show hyperactivation of left M1 gyrus (PwP_onMed_ > HC) and hypoactication of ipsilateral postcentral gyrus (PwP_onMed_ < HC). These two regions are known to be involved in both motor processing and somatosensory function, such as tactile and proprioceptive processing (Pleger et al. 2003). Their opposite activation changes point to dysfunctional bi-hemispheric integration of sensorimotor networks which might be associated with enhanced inhibition from the other hemisphere in the presence of overcompensated medication effect.

### The possible mechanism of levodopa-related over-compensation

L-dopa treatment can improve bradykinesia, but has a risk of inducing drug-related involuntary movements (Poewe et al. 1988; Thanvi et al. 2007). Yet the underlying mechanism has not been fully clarified (see review Vitek 2002; Anderson et al. 2017; Muthuraman et al. 2017). In addition to the restorative upregulation of striatocortical network, we showed hyperactivation in SMA and M1 in medicated patients, which may be associated with the mechanism of potential development of LID and/or uncertain vasomotor effect in the cortical vasculature (Haslinger et al. 2001). Interestingly, this cortical hyperactivation was not revealed in the previous meta-analysis (Herz et al. 2014a). Nevertheless, our findings are supported by an acute levodopa administration study in drug-naive PD patients showing increased SMA functional connectivity from suppressed state as indexed by resting state BOLD signal fluctuations (Esposito et al. 2013). Concordant imaging studies also reported a predominant levodopa effect in the SMA and its neighbouring pre-SMA (Deiber et al. 1999; Playford et al. 1992). Additionally, we identified a consistent overshoot of activation in the left M1 in PwP_onMed_. This may corroborate the levodopa-related over-compensatory effects in the sensorimotor networks, including the primary motor cortex (Cerasa et al. 2012; Herz et al. 2014b).

### The special role of premotor cortex in PD

The SMA has been commonly reported as the region involved in motor-related processes. Yet there are some discrepancies regarding whether this area becomes hypofunctional (Asanuma et al. 2006; Esposito et al. 2013) or hyperfunctional (Haslinger et al. 2001; Poisson et al. 2013; Samuel et al. 1997; Taniwaki et al. 2013) in PD compared to HC during motor task performance. In the meta-analysis, the SMA exhibits reduced activation during the symptomatic stages of PD as we have described above. Moreover, it is the only location showing significant negative correlation to the clinical severity as measured by the UPDRS-III motor scores, which is not revealed in previous findings (Herz et al. 2014a ). Interestingly, few studies of PD gene carriers suggest hyperactivation of preSMA during the presymptomatic phase (Buhmann et al. 2005; B. F. van Nuenen et al. 2009a, b). This evidence may point to a varying role of the SMA during disease progression in the premotor phase and as the motor symptoms deteriorate. Therefore, the heterogeneity of disease severity and disease developing phase in different patient populations may contribute to reported discrepancies in SMA activity levels. Such controversies could also be explained by its role in self-initiated movement in patients but not in controls (see supplementary discussions in SI-1). Fig. 8 summarizes the factors found to modify SMA/medial frontal gyrus activity in PD. Previous Fluorodeoxyglucose18 PET quantification studies have also commonly demonstrated hypoactivation of the SMA in PwP versus HC (Asanuma et al. 2006; Eidelberg et al. 1994; Huang et al. 2007; Ma et al. 2007). This defect can be interpreted as the consequence of the reduction in the positive efferent signal sent from the basal ganglia-thalamo-cortical motor circuit secondary to striatal dopamine depletion (Delong 1990). Interestingly, we found that SMA also showed hyperactivation after taking dopaminergic medication relative to HC, which suggests that levodopa may boost the exhausted compensatory function of SMA motor co-activation. However, drug-induced SMA overactivation was linked with a complex abnormal pattern of contralateral motor hyper- and ipsilateral sensory hypoactivation reflecting functional network disorganization that may lead to LID (Breakefield et al. 2008; Kojovic et al. 2012).

## Limitations and future directions

Every meta-analysis carries over the limitations of the included primary studies. Hence, the present study could not distinguish the motor activation patterns in drug-naive patients from those in patients under long-term treatment because of the limited reports. We can thus not exclude that residual effects of the sustained long-term use of dopaminergic drug might have affected the off medication state. Also, the type of dopaminergic replacement was only partly reported in the medication-effect studies. The second limitation is the very small sample size of studies on the brain activation pattern specific to subtypes of motor impairment due to the recognized underrepresentation of postural instability and gait disorder patients in imaging studies. Thirdly, this study examined the lateralization of motor brain activity pattern in HC and PD. We showed more activation in contralateral hemispheres albeit bilateral occurrences in different MAC analyses (also see SI-2 and Fig. S5 for more discussion). Our lateralised disease-specific pattern and medication effect may also indicate consequence attributing to a loss of compensation. However, due to the lack of consistency of the disease laterality, it is not possible to investigate whether there is a compensatory effect from the less degenerated side. In the current study, we focused on the spatial activation pattern of motor tasks with patients’ right hands. It would be also interesting to reveal the spatial representation of lower limbs, which is related to the clinical staging.

Despite previous studies having demonstrated the common activation regions shared by motor imagery and execution (Baik et al. 2014; Dominey et al. 1995), we decided not to include motor imagery studies as they involve additional complex cognitive processes. The current data is further limited in providing confirmatory evidence on the role of the cerebellum in PD as not all studies achieve complete brain coverage. Moreover, we studied motor executive abnormality using task fMRI and PET but did not include resting state BOLD fMRI and ASL fMRI studies. Functional connectivity changes are increasingly recognised in PD (Baik et al. 2014; Hacker et al. 2012; Helmich et al. 2010; H. Yu et al. 2007) with cumulating evidence for dysfunction of the entire intrinsic distribution of the sensorimotor network (see review (Tahmasian et al. 2015)). Finally, further and more neuroimaging studies in people at high risk of developing PD, such as carriers of mutated genes, may be able to provide more consistent insight into the putative neural compensatory mechanisms underlying the motor preservation during the pre-symptomatic stage of PD.

## Conclusions

Our CBMA reports the consistent activation pattern of known cortical and subcortical motor networks in normal volunteers and people with clinical Parkinson’s. We demonstrate striatal and SMA hypoactivation as the core abnormality in Parkinson’s off medication during motor tasks, which appears to be reversed by restorative dopaminergic effect. Previous inconsistent reports can be explained by a complex role of SMA/pre-SMA activation changes in task- and medication-specific variation in intention. Importantly, our meta-analysis reveals an activity reduction in SMA during STN deep brain stimulation, which might also underpin L-dopa related overcompensation. We recommend more attention to be drawn to the neuronal network alterations by combining resting and active state in PD. Also, further high quality neuroimaging motor studies should focus on the neural compensatory mechanisms underlying the motor preservation in people at high risk of developing PD or in the premotor stage.

## Electronic supplementary material

ESM 1 (DOCX 758 kb)
