# Supplementary material for: Coordinate based meta-analysis of motor functional imaging in Parkinson’s: disease-specific patterns and modulation by dopamine replacement and deep brain stimulation
Source: Brain Imaging Behav. 2019 Feb 27;14(4):1263–80. doi: 10.1007/s11682-019-00061-3 (PMC7381438; doi:10.1007/s11682-019-00061-3)
Supplement: Supplementary file 1 — (DOCX 758 kb) [file 11682_2019_61_MOESM1_ESM.docx]

## SI-1.The neuronal correlates of intentional movement

PwP usually exploit external cues to help them improve performance of motor initiation [(Almeida, Wishart, & Lee, 2002; Lim et al., 2005) for example]. However, controversial results have been shown in previous studies that investigated the neuronal activity relating to self-initiation in PwP (Buhmann et al., 2003; Eckert, Peschel, Heinze, & Rotte, 2006; Jenkins et al., 1992; Playford et al., 1992; Sabatini et al., 2000; Samuel et al., 1997). Different components underlying this process might account for this dispute (Hoffstaedter, Grefkes, Zilles, & Eickhoff, 2013). Therefore, we specifically looked for the motor function studies that examined the neuronal mechanism underlying 1) a component associated to what action to perform, for instance, whether to tap fingers at fixed sequence or at random; 2) a component about when to execute an action, taking self-initiated (SI) versus externally triggered (ET) conditions for example in both HC and PwP. Possibly due to the limited numbers of studies for component 1 and/or different levels of task complexity (Nachev, Kennard, & Husain, 2008), we failed to see the differential activation pattern or strength in the brain. In terms to component 2, no consistent region was found when comparing SI and ET for HC in neither MAC nor CMA analysis, indicating that similar timing networks might be recruited to support these two rhythmic movements in *healthy groups* (Cerasa et al., 2006). Nevertheless, the converging results from MAC analyses showed hyper-activation in precuneus and cuneus in external condition in *PwP_offMed_*, suggesting that the patients employed the related sensory modalities to compensate the motor impairment, such as visualizing an external cue to initiate movement by activating these regions more. This is consistent with their role of coordination and attention to motor (Cavanna & Trimble, 2006; Wenderoth, Debaere, Sunaert, & Swinnen, 2005).

On the other hand, our CMA localised hyper-activated medial frontal region (SMA and/or pre-SMA) in PwP_offMed_ >HC for SI>ET. This conjunctional result might be caused by interaction effect of disease and initiation. But combining the null significant spatial pattern difference for PwP_offMed_>HC during the *external cued condition* from our MAC analysis, we postulate that the relative higher medial frontal region activation in PwP_offMed_ >HC× SI>ET is more likely to be the compensatory role of more activated SMA to maintain self-initiation in PwP. Noticeably, an effect of motor learning is unlikely to be the attribution to this increased activation in SMA (Carbon et al., 2003; Nakamura et al., 2001) as the studies recruited for this analysis all claimed that their subjects over-learned the task prior to scanning.

Given the increase of spatial resolution of fMRI that facilitates us to map a refinement of motor-related functional organization, we further categorised this area according to the locations and boundaries of pre-SMA and SMA (both are included in Brodmann 6) defined in one previous meta-analysis (Mayka, Corcos, Leurgans, & Vaillancourt, 2006). We realized that the peak coordinates of the clusters are more in the vicinity of pre-SMA or anterior part of SMA. In the same vein, one previous fMRI study showed differential activation in rostral and caudal SMA(Sabatini et al., 2000) after comparing the akinetic PwP and HC while they were implementing a sequential task, which may be corresponding to the pre-SMA and SMA proper in primate studies (Matsuzaka, Aizawa, & Tanji, 1992; Rizzolatti, Luppino, & Matelli, 1998; Tanji, 1994) and other human studies involving whole-body gymnastic movement (Zentgraf et al., 2005). This is consistent with more recent understanding that preSMA has a greater role in motor preparation, initiation and motor attention, whereas SMA proper is more engaged with motor performance and execution based on structural and physiological foundations (Mayka et al., 2006; Nachev et al., 2008; Picard & Strick, 1996; Rouiller et al., 1994; Schell & Strick, 1984). In addition, one previous study on asymptomatic carriers of mutant Parkin allele also showed elevation of activation during motor task in rostral SMA during internal movement selection compared with externally guided action (Buhmann et al., 2005).

## SI-2. Lateralization of motor brain activity pattern in PD

It is well accepted that the neurons in contralateral hemisphere fire more when the opposite side of the body is in action and this has been confirmed by previous individual neuroimaging studies with HC (Catalan, Honda, Weeks, Cohen, & Hallett, 1998; Mattay et al., 1998; Solodkin, Hlustik, Noll, & Small, 2001). Indeed, the results uncovered by the present meta-analysis showed more activation in left hemispheres. This phenomenon may be explained that out of all the studies, we merged 4 studies that asked their participants to use both sides of upper limbs simultaneously, and 2 with most-affected side of the body. However, even during unilateral movement, a recent study on the lateralization of brain activity pattern has shown its disruption in PD using effective dynamic connectivity analysis (Wu, Hou, Hallett, Zhang, & Chan, 2015). Supporting this previous finding, after applying CBMA to all-HC and all-PwP group with right-hand task studies *only*, respectively, we also detected less laterality in M1 in patient group (Figure S5). This may provide imaging evidence for some abnormal involuntary movement in PD, such as mirror movement (Borgheresi et al., 2010; Espay, Morgante, Gunraj, Chen, & Lang, 2006) or normal bilateral movement coordination, which may require inhibition of the unnecessary activity of the other hand (Carson, 2005).


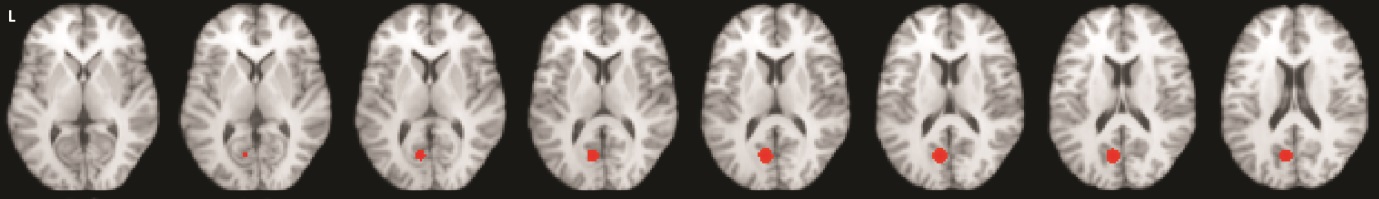


**Figure S1.** By applying the MAC to the contrast outcomes across investigations of the neuronal correlates of SI and ET movement in both HC and PwP_offMed_, we found that out of the 24 foci, left cuneus (Talairach atlas-based x, y, z: -5.1 -72.4 15.3; *p=*0.000021*)* and left precuneus (Talairach atlas-based x, y, z: -5.2 -69 18.4) showed significant stronger activation for ET>SI only in patients’ group. No region presented significant result for SI>ET.

**Figure S2**: The frequency of reported articles. The regions with the biggest difference of reported frequency reported on HC, PwP_offMed_ and PwP_onMed_, where zero frequency indicates no report from that particular group.


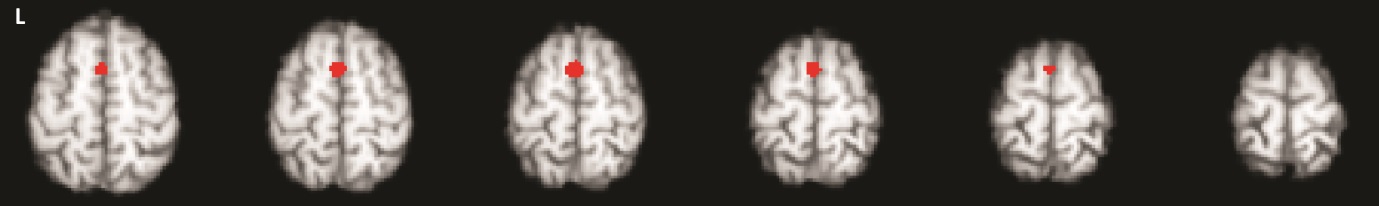


**Figure S3**: Increased medial frontal activation in self-paced vs. external cued motor task using contrast meta-analysis (SI>ET in PwP_offMed_>HC group, n=98 patients vs. n=148 HC, n= 55 experiments, FCDR corrected, P<0.05).


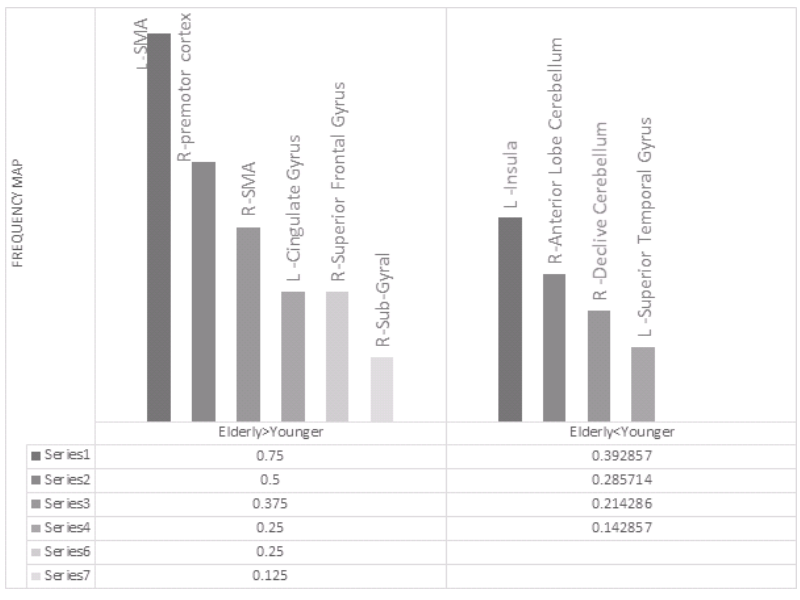


**Figure S4.** The frequency of regions that showed age difference. Left: the regions that contributed significantly to the elderly>younger group; Right: the areas the younger population showed significant more activation than the elderly group. Their reported frequencies were sorted in descending order and the values were listed below each chart.


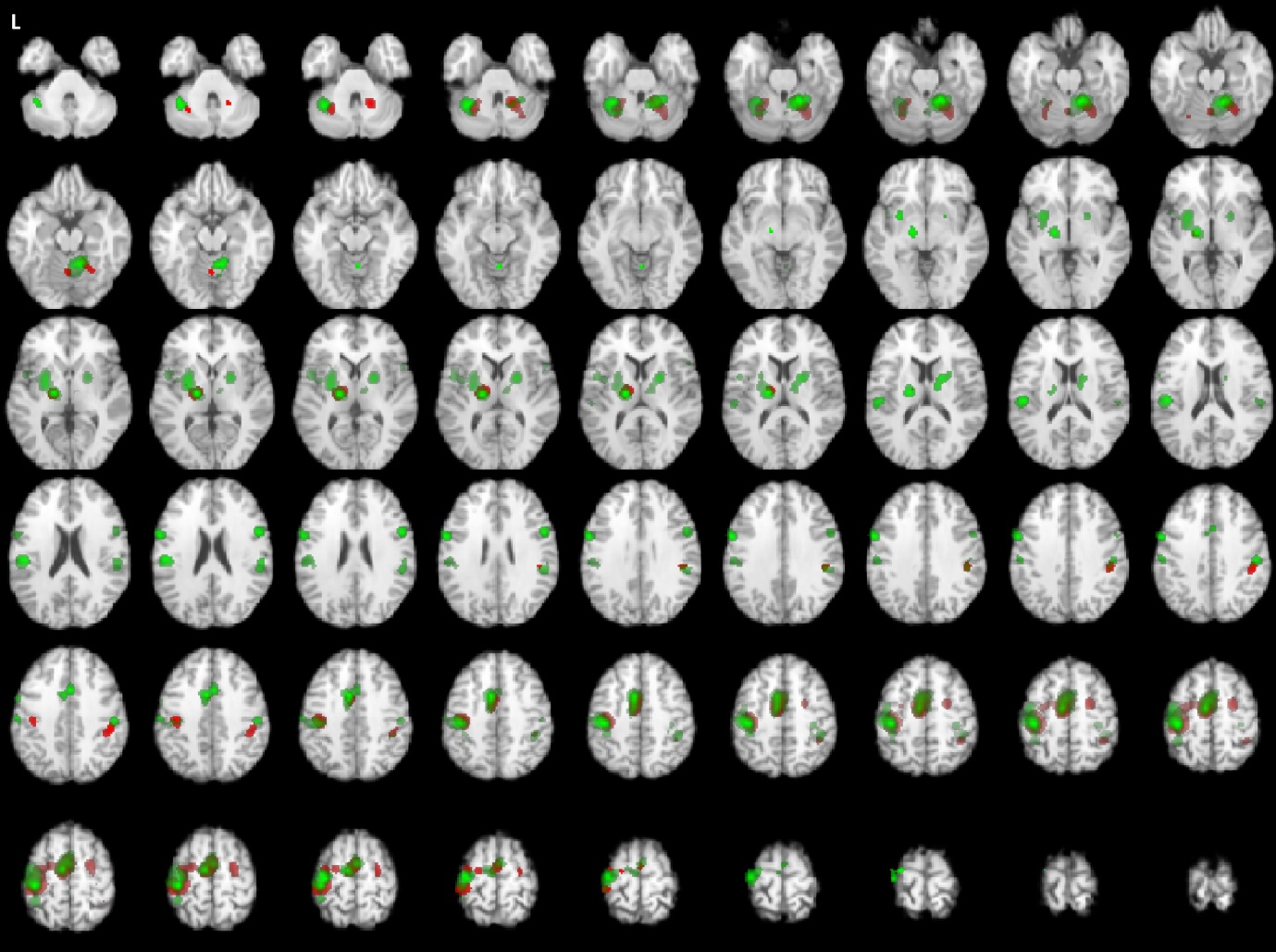


**Figure S5.** The lateralization of brain activity pattern during right-hand motor task in HC(green) and PwP(red).

**Table S1. More detailed study information recruited in our meta-analysis**

| *Study* | *#HC* | *#PD* | *Foci* | *Modality* | *Multiple comparison Corrected* | *Condition* | *mean Age PwP* | *mean*  *Age*  *HC* |
| --- | --- | --- | --- | --- | --- | --- | --- | --- |
| SAMUEL  1998 | 5 | - | 24 | fMRI | No | HC | - | 32 |
| RIECKER  2006 | 10  elderly | - | 14 | fMRI | Yes | HC | - | 66 |
|  | 10 young |  |  |  |  |  |  | 23 |
| TRACY  2001 | 5 | - | 18 | fMRI | Yes | HC | - | 23.6 |
| BEDARD  2009 | 15 | - | 19 | fMRI | Yes | HC | - | 19-34 |
| CHAN  2006 | 10 | - | 7 | fMRI | Yes | HC | - | 22.9 |
| HARRINGTON  2000 | 15 | - | 10 | fMRI | No | HC | - | 28.8 |
| CUNNINGTON  2002 | 12 | - | 19 | fMRI | Yes | HC | - | 23.9 |
| HASLINGER  2002 | 8 | - | 26 | fMRI | No | HC | - | 26 |
| JOA2012 | 19 | - | 18 | fMRI | Yes | HC | - | 28 |
| KUHTZ-BUSCHBECK  2003 | 12 | - | 33 | fMRI | Yes | HC | - | 24 |
| KUHTZ-BUSCHBECK  2008 | 14 | - | 78 | fMRI | Yes | HC | - | 26 |
| LEHERICY  2006 | 12 | - | 46 | fMRI | Yes | HC | - | 22.9 |
| LOUBINOUX  2001 | 21 | - | 25 | fMRI | Yes | HC | - | 41 |
| MEINDL  2008 | 6 | - | 40 | fMRI | No | HC | - | 32.8 |
| ROWE  2002 | 15 | - | 4 | fMRI | Yes | HC | - | 29 |
| SCHMID  2004 | 6 | - | 42 | fMRI | Yes | HC | - | 27 |
| WARD  2003 | 26 | - | 79 | fMRI | Yes | HC | - | 48.3 |
| YOO  2005 | 8 | - | 17 | fMRI | No | HC | - | 35.1 |
| JIANG  2004 | 8 | - | 24 | fMRI | No | HC | - | 22 |
| STOODLEY  2012 | 9 | - | 20 | fMRI | Yes | HC | - | 25 |
| WENDEROTH  2005 | 10 | - | 27 | fMRI | Yes | HC | - | 25 |
| JENKINS  1997 | 6 | - | 15 | PET | Yes | HC | - | 37.3 |
| AOKI  2005 | 10 | - | 8 | PET | Yes | HC | - | 22.2 |
| BOECKER  1998 | 7 | - | 20 | PET | Yes | HC | - | 32 |
| GOERRES  1998 | 6 | - | 54 | PET | No | HC | - | 65 |
| JENKINS  2000 | 6 | - | 20 | PET | Yes | HC | - | 32 |
| JUEPTNER  1997 | 12 | - | 10 | PET | No | HC | - | 25.5 |
| KAWASHIMA  1996 | 8 | - | 25 | PET | No | HC | - | 19-21 |
| KINOSHITA  2000 | 10 | - | 18 | PET | Yes | HC | - | 22.7 |
| MIMA  1999 | 6 | - | 9 | PET | Yes | HC | - | 22.5 |
| SADATO  1997 | 9 | - | 62 | PET | Yes | HC | - | 22.3 |
| TAKASAWA  2003 | 7 | - | 6 | PET | Yes | HC | - | 23-24 |
| EHRSSON  2000 | 8 | - | 14 | PET | Yes | HC | - | 24-27 |
| PARKINSON  2009 | 11 | - | 11 | fMRI | Yes | HC | - | 28 |
| MULLLER  2009 | 11 | - | 12 | fMRI | Yes | HC | - | 34.5 |
| HABAS  2008 | 7 | - | 33 | fMRI | No | HC | - | 25 |
| KAWASHIMA  1998 | 8 | - | 27 | PET | Yes | HC | - | 20-24 |
| HASLINGER  2001 | 8 | - | 6 | fMRI | No | HC | - | 54.4 |
| ECKERT  2006 | 9 | - | 7 | fMRI | Yes | HC | - | 60.6 |
| TANIWAKI  2013 | 12 | - | 35 | fMRI | No | HC | - | 62 |
| KRAFT  2009 | 12 | - | 20 | fMRI | No | HC | - | 53 |
| MALLOL  2007 | 11 | - | 17 | fMRI | No | HC | - | 61.9 |
| CERESA  2006 | 11 | - | 9 | fMRI | Yes | HC | - | 63.4 |
| YU  2007 | 8 | - | 51 | fMRI | Yes | HC | - | 59.5 |
| KALMAR  2011 | 5 | - | 5 | fMRI | Yes | HC | - | 26-35 |
| PINTO  2011 | 15 | - | 13 | fMRI | Yes | HC |  | 55 |
| ROWE  2002 | 18 | - | 10 | fMRI | No | HC | - | 62 |
| KWON  2011 | 15 | - | 16 | fMRI | No | HC | - | 25.5 |
| RAPOSO  2009 | 22 | - | 7 | fMRI | Yes | HC | - | 23 |
| VRY  2012 | 22 | - | 22 | fMRI | Yes | HC | - | 31 |
| LOIBL  2011 | 17  elderly | - | 95 | fMRI | Yes | HC | - | 66.7 |
|  | 18 young |  |  |  |  |  |  | 25.4 |
| ZAPPAROLI  2013 | 24  elderly | - | 64 | fMRI | Yes | HC | - | 60 |
|  | 24 young |  |  |  |  |  |  | 27 |
| HANAKAWA  2003 | 10 | - | 14 | fMRI | Yes | HC | - | 32 |
| SAMUEL  2001 | 6 | - | 14 | PET | No | HC | - | 55 |
| THOBOIS  2000 | 8 | - | 20 | PET | Yes | HC | - | 54 |
| SEITZ  1997 | 8 | - | 8 | PET | No | HC | - | 28 |
| HASLINGER  2001 | - | 8 | 5 | fMRI | No | PDoff | 54.4 | - |
| ECKERT  2006 | - | 9 | 10 | fMRI | Yes | PDoff | 60.6 | - |
| TANIWAKI  2013 | - | 12 | 22 | fMRI | No | PDoff | 62 | - |
| KRAFT  2009 | - | 12 | 14 | fMRI | No | PDoff | 53 | - |
| MALLOL  2007 | - | 13 | 7 | fMRI | No | PDoff | 61.9 | - |
| CERESA  2006 | - | 10 | 20 | fMRI | Yes | PDoff | 63.4 | - |
| YU  2007 | - | 8 | 31 | fMRI | Yes | PDoff | 59.5 | - |
| KALMAR  2011 | - | 5 | 13 | fMRI | Yes | PDoff | 26-35 | - |
| PINTO  2011 | - | 9 | 6 | fMRI | Yes | PDoff | 55 | - |
| JENKINS  1992 | - | 8 | 2 | PET | Yes | PDoff | 32 | - |
| MAILLET  2012 | - | 12 | 13 | fMRI | Yes | PDoff | 60 | - |
| ROWE  2002 | - | 18 | 9 | fMRI | No | PDoff | 62 | - |
| SAMUEL  2001 | - | 6 | 9 | PET | No | PDoff | 55 | - |
| THOBOIS  2000 | - | 8 | 24 | PET | Yes | PDoff | 54 | - |
| HASLINGER  2001 | - | 8 | 5 | fMRI | No | PDon | 54.4 | - |
| ECKERT  2006 | - | 9 | 9 | fMRI | Yes | PDon | 60.6 | - |
| KRAFT  2009 | - | 12 | 18 | fMRI | No | PDon | 53 | - |
| JENKINS  1992 | - | 8 | 3 | PET | Yes | PDon | 53.9 | - |
| MAILLET  2012 | - | 12 | 2 | fMRI | Yes | PDon | 60 | - |
| HASLINGER  2001 | 8 | 8 | 10 | fMRI | No | PDon vs. PDoff | 60.8 | 54.4 |
| ECKERT  2006 | 9 | 9 | 4 | fMRI | Yes | PDon vs. PDoff | 63.3 | 60.6 |
| FEIGIN  2003 | - | 7 | 2 | PET | No | PDon vs. PDoff | 60.1 | - |
| KRAFT  2009 | 12 | 12 | 8 | fMRI | Yes | PDon vs. PDoff | 60.8 | 53 |
| HUGHES  2010 | 15 | 16 | 1 | fMRI | No | PDon vs. PDoff | 63.9 | 66.5 |
| MARTINU  2012 | 14 | 12 | 30 | fMRI | Yes | PDon vs.  PDoff | 63 | 61.7 |
| PAYOUX  2010 | 10 | 8 | 1 | PET | Yes | PDon vs. PDoff | 62 | 67 |
| FEIGIN  2002 | - | 7 | 4 | PET | Yes | PDon vs. PDoff | 59.4 | - |
| HASLINGER  2001 | 8 | 8 | 8 | fMRI | No | PDon vs. HC | 60.8 | 54.4 |
| ECKERT  2006 | 9 | 9 | 9 | fMRI | Yes | PDon vs. HC | 63.3 | 60.6 |
| KRAFT  2009 | 12 | 12 | 12 | fMRI | Yes | PDon vs. HC | 60.8 | 53 |
| HUGHES  2010 | 15 | 16 | 15 | fMRI | No | PDon vs. HC | 63.9 | 66.5 |
| MARTINU  2012 | 14 | 12 | 30 | fMRI | Yes | PDon vs.  HC | 63 | 61.7 |
| HASLINGER  2001 | 8 | 8 | 7 | fMRI | No | PDoff vs. HC | 60.8 | 54.4 |
| BUHMANN  2003 | 10 | 8 | 2 | fMRI | No | PDoff vs. HC | 54 | 57 |
| MARTINU  2012 | 14 | 12 | 30 | fMRI | Yes | PDoff vs.  HC | 63 | 61.7 |
| ECKERT  2006 | 9 | 9 | 18 | fMRI | Yes | PDoff vs. HC | 63.3 | 60.6 |
| CAPARONI  2013 | 11 | 11 | 26 | fMRI | No | PDoff vs. HC | 65 | 65.1 |
| TANIWAKI  2013 | 12 | 12 | 7 | fMRI | No | PDoff vs. HC | 63.7 | 62 |
| KRAFT  2009 | 12 | 12 | 25 | fMRI | Yes | PDoff vs. HC | 60.8 | 53 |
| GONZA´LEZ-GARCI´A  2011 | 10 | 17 | 13 | fMRI | No | PDoff vs. HC | 64.4 | - |
| MALLOL  2007 | 11 | 13 | 13 | fMRI | No | PDoff vs. HC | 64.9 | 61.9 |
| PRODOEHL  2010 | 20 | 20 | 18 | fMRI | Yes | PDoff vs. HC | 57.9 | 58.3 |
| WU  2010 | 15 | 15 | 35 | fMRI | Yes | PDoff vs. HC | 59.73 | 60.3 |
| TESSA  2013 | 10 | 11 | 6 | fMRI | Yes | PDoff vs. HC | 68 | 64 |
| CERESA  2006 | 11 | 10 | 11 | fMRI | Yes | PDoff vs. HC | 63.7 | 63.4 |
| POISSON  2013 | 10 | 6 | 13 | fMRI | No | PDoff vs. HC | 65 | 53.6 |
| YU  2007 | 8 | 8 | 13 | fMRI | No | PDoff vs. HC | 59.4 | 59.5 |
| PINTO  2011 | 15 | 9 | 6 | fMRI | No | PDoff vs. HC | 59 | 55 |
| HUGHES  2010 | 15 | 16 | 1 | fMRI | No | PDoff vs. HC | 63.9 | 66.5 |
| ROWE  2002 | 12 | 12 | 0 | fMRI | No | PDoff vs. HC | 62 | 62 |
| WU  2005 | 10 | 26 | 12 | fMRI | No | PDoff vs. HC | 61.2 | 61.8 |
| TOXOPEUS  2012 | 18 | 12 | 79 | fMRI | No | PDoff vs. HC | 58.1 | 58.7 |
| BURCIU  2015 | 20 | 20 | 20 | fMRI | Yes | PDoff vs. HC | 65.8 | 64.8 |
| VAN DER STOUWE  2015 | 13 | 12 | 15 | fMRI | No | PDoff vs. HC | 38.9 | 38.7 |
| WU  2015 | 26 | 26 | 7 | fMRI | Yes | PDoff vs. HC | 58.96 | 58.92 |
| CATALAN  1999 | 13 | 13 | 7 | PET | No | PDoff vs. HC | 52.5 | 51.7 |
| JAHANSHAHI  1995 | 6 | 6 | 39 | PET | Yes | PDoff vs. HC | 64 | 64.8 |
| PAYOUX  2010 | 10 | 8 | 3 | PET | Yes | PDoff vs. HC | 62 | 67 |
| SAMUEL  1997 | 6 | 6 | 17 | PET | No | PDoff vs. HC | 70.2 | 64.3 |
|  |  |  |  |  |  |  |  |  |
|  |  |  |  |  |  |  |  |  |

Table S2: Summary of articles of deep brain stimulation in subthalamus nuclei

| **Papers** | **Med status** | | | **Side of STN** | | | **# Subj** | **During movement** | | **During rest** | |
| --- | --- | --- | --- | --- | --- | --- | --- | --- | --- | --- | --- |
| Author  Publication Year | On-med | Off-med | Unilateral-STN | | Mixed sides | Bilateral-STN |  | Increase | Decrease | Increase | Decrease |
| CILIA2008 | Y |  |  | |  | Y | 40 |  |  | Y (9) | Y (21) |
| HILKER2003 |  | Y |  | |  | Y | 8 |  |  | Y (11) | Y (1) |
| KARIMI2008 |  | Y |  | |  | Y | 31 |  |  | Y (3) | Y (1) |
| GEDAY2009 |  | Y |  | |  | Y | 10 |  |  | Y (7) | Y (6) |
| HERSHEY2003 |  | Y |  | |  | Y | 13 |  |  | Y (2) | Y (9) |
| JECH2001 | Y |  |  | | Y  (3L, 1R) |  | 4 |  |  | Y (12) |  |
| PAYOUX2004 |  | Y |  | |  | Y | 7 |  | Y (5) |  | Y (5) |
| THOBOIS2002 |  | Y | Y (left) | |  |  | 7 | Y (5) | Y (3) |  |  |
| LIMOUSIN1997 |  | Y |  | |  | Y | 12 | Y (3) |  |  | Y (3) |
| ASANUMA2006 |  | Y |  | |  | Y | 9 |  |  | Y (2) | Y (8) |
| GRAFTON2006 |  | Y |  | | Y  (5L, 1R) |  | 6 | Y (6) | Y (9) | Y (15) |  |
| STRAFELLA2003 |  | Y | Y | | Y  (Ipsilateral side of right hand) | Y | 5 | Y (22) |  |  |  |
| CEBALLOS-BAUMANN1999 |  | Y |  | | Y  (contralateral side of right hand) |  | 8 | Y (3) | Y (2) | Y (7) | Y (4) |

Increase: STNDBS-On > STNDBS-off

Decrease: STNDBS-On < STNDBS-off

Table S3: Summary of dopaminergic replacement therapy in the on-medication studies

| *Publication* | *DRT type* | *L-dopa equivalent dose* |
| --- | --- | --- |
| HASLINGER | 1 levodopa; 2 dopamine agonist | x |
| ECKERT | oral levodopa | 311.1+/-42.0 mg |
| KRAFT | oral administration of resolved levodopa+carbidopa | 200 mg levodopa + 50 mg carbidopa |
| JENKINS | apomorphine infusion | 4.41+/-2.3 mg/h |
| MAILLET | levodopa | 303+/-79 mg |
| FEIGIN, 2003 | levodopa infusion | 67.1+/-25.6 mg/h |
| HUGHES | oral levodopa not specific | 1271.1+/-527.8 mg |
| MARTINU | 1, levodopa; 2, Catechol-O-methyl transferase inhibitor; 3, Monoamine oxidase B inhibitor; 4, dopamine agonist | x |
| PAYOUX | oral levodopa not specific | 767 +/- 400 mg |
| FEIGIN, 2002 | levodopa infusion | 67.1+/-25.6 mg/h |

x: no detail was reported.

Reference:

Almeida, Q. J., Wishart, L. R., & Lee, T. D. (2002). Bimanual coordination deficits with Parkinson's disease: the influence of movement speed and external cueing. *Mov Disord, 17*(1), 30-37.

Borgheresi, A., Espay, A. J., Giovannelli, F., Vanni, P., Zaccara, G., & Cincotta, M. (2010). Congenital mirror movements in Parkinson's disease: clinical and neurophysiological observations. *Mov Disord, 25*(10), 1520-1523. doi:10.1002/mds.23142

Buhmann, C., Binkofski, F., Klein, C., Buchel, C., van Eimeren, T., Erdmann, C., . . . Siebner, H. R. (2005). Motor reorganization in asymptomatic carriers of a single mutant Parkin allele: a human model for presymptomatic parkinsonism. *Brain, 128*(Pt 10), 2281-2290. doi:10.1093/brain/awh572

Buhmann, C., Glauche, V., Sturenburg, H. J., Oechsner, M., Weiller, C., & Buchel, C. (2003). Pharmacologically modulated fMRI--cortical responsiveness to levodopa in drug-naive hemiparkinsonian patients. *Brain, 126*(Pt 2), 451-461.

Carbon, M., Ghilardi, M. F., Feigin, A., Fukuda, M., Silvestri, G., Mentis, M. J., . . . Eidelberg, D. (2003). Learning networks in health and Parkinson's disease: reproducibility and treatment effects. *Hum Brain Mapp, 19*(3), 197-211. doi:10.1002/hbm.10115

Carson, R. G. (2005). Neural pathways mediating bilateral interactions between the upper limbs. *Brain Research Reviews, 49*(3), 641-662. doi:10.1016/j.brainresrev.2005.03.005

Catalan, M. J., Honda, M., Weeks, R. A., Cohen, L. G., & Hallett, M. (1998). The functional neuroanatomy of simple and complex sequential finger movements: a PET study. *Brain, 121 ( Pt 2)*, 253-264.

Cavanna, A. E., & Trimble, M. R. (2006). The precuneus: a review of its functional anatomy and behavioural correlates. *Brain, 129*(Pt 3), 564-583. doi:10.1093/brain/awl004

Cerasa, A., Hagberg, G. E., Peppe, A., Bianciardi, M., Gioia, M. C., Costa, A., . . . Sabatini, U. (2006). Functional changes in the activity of cerebellum and frontostriatal regions during externally and internally timed movement in Parkinson's disease. *Brain Res Bull, 71*(1-3), 259-269. doi:10.1016/j.brainresbull.2006.09.014

Eckert, T., Peschel, T., Heinze, H. J., & Rotte, M. (2006). Increased pre-SMA activation in early PD patients during simple self-initiated hand movements. *J Neurol, 253*(2), 199-207. doi:10.1007/s00415-005-0956-z

Espay, A. J., Morgante, F., Gunraj, C., Chen, R., & Lang, A. E. (2006). Mirror movements in Parkinson's disease: effect of dopaminergic drugs. *J Neurol Neurosurg Psychiatry, 77*(10), 1194-1195. doi:10.1136/jnnp.2005.086892

Hoffstaedter, F., Grefkes, C., Zilles, K., & Eickhoff, S. B. (2013). The "What" and "When" of Self-Initiated Movements. *Cereb Cortex, 23*(3), 520-530. doi:10.1093/cercor/bhr391

Jenkins, I. H., Fernandez, W., Playford, E. D., Lees, A. J., Frackowiak, R. S., Passingham, R. E., & Brooks, D. J. (1992). Impaired activation of the supplementary motor area in Parkinson's disease is reversed when akinesia is treated with apomorphine. *Ann Neurol, 32*(6), 749-757. doi:10.1002/ana.410320608

Lim, I., van Wegen, E., de Goede, C., Deutekom, M., Nieuwboer, A., Willems, A., . . . Kwakkel, G. (2005). Effects of external rhythmical cueing on gait in patients with Parkinson's disease: a systematic review. *Clin Rehabil, 19*(7), 695-713.

Matsuzaka, Y., Aizawa, H., & Tanji, J. (1992). A Motor Area Rostral to the Supplementary Motor Area (Presupplementary Motor Area) in the Monkey - Neuronal-Activity during a Learned Motor Task. *J Neurophysiol, 68*(3), 653-662.

Mattay, V. S., Callicott, J. H., Bertolino, A., Santha, A. K., Van Horn, J. D., Tallent, K. A., . . . Weinberger, D. R. (1998). Hemispheric control of motor function: a whole brain echo planar fMRI study. *Psychiatry Res, 83*(1), 7-22.

Mayka, M. A., Corcos, D. M., Leurgans, S. E., & Vaillancourt, D. E. (2006). Three-dimensional locations and boundaries of motor and premotor cortices as defined by functional brain imaging: A meta-analysis. *Neuroimage, 31*(4), 1453-1474. doi:10.1016/j.neuroimage.2006.02.004

Nachev, P., Kennard, C., & Husain, M. (2008). Functional role of the supplementary and pre-supplementary motor areas. *Nature Reviews Neuroscience, 9*(11), 856-869. doi:10.1038/nrn2478

Nakamura, T., Ghilardi, M. F., Mentis, M., Dhawan, V., Fukuda, M., Hacking, A., . . . Eidelberg, D. (2001). Functional networks in motor sequence learning: abnormal topographies in Parkinson's disease. *Hum Brain Mapp, 12*(1), 42-60.

Picard, N., & Strick, P. L. (1996). Motor areas of the medial wall: A review of their location and functional activation. *Cerebral Cortex, 6*(3), 342-353. doi:DOI 10.1093/cercor/6.3.342

Playford, E. D., Jenkins, I. H., Passingham, R. E., Nutt, J., Frackowiak, R. S., & Brooks, D. J. (1992). Impaired mesial frontal and putamen activation in Parkinson's disease: a positron emission tomography study. *Ann Neurol, 32*(2), 151-161. doi:10.1002/ana.410320206

Rizzolatti, G., Luppino, G., & Matelli, M. (1998). The organization of the cortical motor system: new concepts. *Electroencephalography and Clinical Neurophysiology, 106*(4), 283-296. doi:Doi 10.1016/S0013-4694(98)00022-4

Rouiller, E. M., Babalian, A., Kazennikov, O., Moret, V., Yu, X. H., & Wiesendanger, M. (1994). Transcallosal connections of the distal forelimb representations of the primary and supplementary motor cortical areas in macaque monkeys. *Exp Brain Res, 102*(2), 227-243.

Sabatini, U., Boulanouar, K., Fabre, N., Martin, F., Carel, C., Colonnese, C., . . . Rascol, O. (2000). Cortical motor reorganization in akinetic patients with Parkinson's disease: a functional MRI study. *Brain, 123 ( Pt 2)*, 394-403.

Samuel, M., Ceballos-Baumann, A. O., Blin, J., Uema, T., Boecker, H., Passingham, R. E., & Brooks, D. J. (1997). Evidence for lateral premotor and parietal overactivity in Parkinson's disease during sequential and bimanual movements. A PET study. *Brain, 120 ( Pt 6)*, 963-976.

Schell, G. R., & Strick, P. L. (1984). The origin of thalamic inputs to the arcuate premotor and supplementary motor areas. *J Neurosci, 4*(2), 539-560.

Solodkin, A., Hlustik, P., Noll, D. C., & Small, S. L. (2001). Lateralization of motor circuits and handedness during finger movements. *Eur J Neurol, 8*(5), 425-434.

Tanji, J. (1994). The Supplementary Motor Area in the Cerebral-Cortex. *Neuroscience Research, 19*(3), 251-268. doi:Doi 10.1016/0168-0102(94)90038-8

Wenderoth, N., Debaere, F., Sunaert, S., & Swinnen, S. P. (2005). The role of anterior cingulate cortex and precuneus in the coordination of motor behaviour. *Eur J Neurosci, 22*(1), 235-246. doi:10.1111/j.1460-9568.2005.04176.x

Wu, T., Hou, Y., Hallett, M., Zhang, J., & Chan, P. (2015). Lateralization of brain activity pattern during unilateral movement in Parkinson's disease. *Hum Brain Mapp, 36*(5), 1878-1891. doi:10.1002/hbm.22743

Zentgraf, K., Stark, R., Reiser, M., Kunzell, S., Schienle, A., Kirsch, P., . . . Munzert, J. (2005). Differential activation of pre-SMA and SMA proper during action observation: effects of instructions. *Neuroimage, 26*(3), 662-672. doi:10.1016/j.neuroimage.2005.02.015
